# Effect of Magnetic Anisotropy on the ^1^H NMR Paramagnetic Shifts and Relaxation Rates of Small Dysprosium(III) Complexes

**DOI:** 10.1021/acs.inorgchem.3c01959

**Published:** 2023-08-21

**Authors:** Charlene Harriswangler, Fátima Lucio-Martínez, Léna Godec, Lohona Kevin Soro, Sandra Fernández-Fariña, Laura Valencia, Aurora Rodríguez-Rodríguez, David Esteban-Gómez, Loïc J. Charbonnière, Carlos Platas-Iglesias

**Affiliations:** † Centro Interdisciplinar de Química e Bioloxía (CICA) and Departamento de Química, Facultade de Ciencias, Universidade da Coruña, 15071 A Coruña, Galicia, Spain; ‡ Equipe de Synthèse Pour l′Analyse (SynPA), Institut Pluridisciplinaire Hubert Curien (IPHC), UMR 7178, CNRS, Université de Strasbourg, ECPM, 25 rue Becquerel, 67087 Strasbourg Cedex, France; § Departamento de Química Inorgánica, Facultad de Ciencias, Universidade de Vigo, As Lagoas, Marcosende, 36310 Pontevedra, Spain; ∥ Departamento de Química Inorgánica, Facultade de Química, Campus Vida, Universidade de Santiago de Compostela, 15782 Santiago de Compostela, Spain

## Abstract

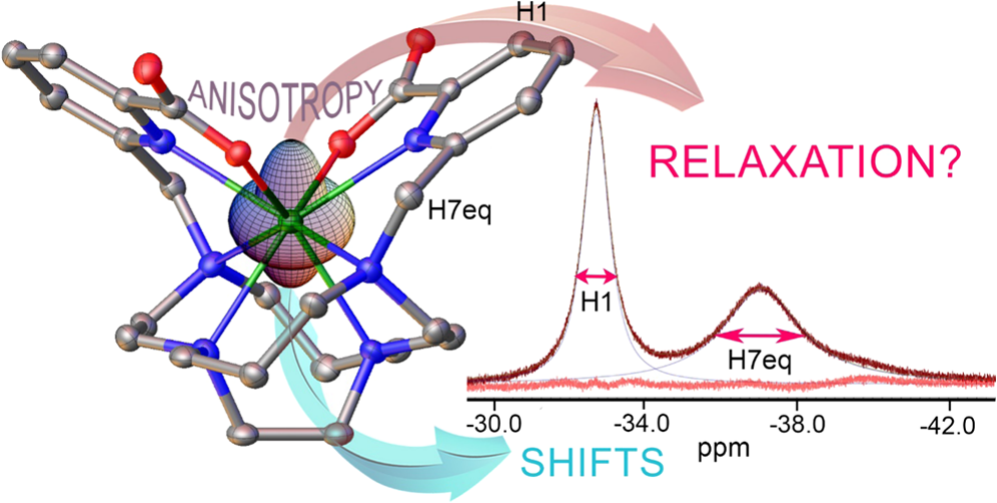

We present a detailed analysis of the ^1^H NMR chemical shifts and transverse relaxation rates of three small Dy(III) complexes having different symmetries (*C*_3_, *D*_2_ or *C*_2_). The complexes show sizeable emission in the visible region due to ^4^F_9/2_ → ^6^H_J_ transitions (*J* = 15/2 to 11/2). Additionally, NIR emission is observed at ca. 850 (^4^F_9/2_ → ^6^H_7/2_), 930 (^4^F_9/2_ → ^6^H_5/2_), 1010 (^4^F_9/2_ → ^6^F_9/2_), and 1175 nm (^4^F_9/2_ → ^6^F_7/2_). Emission quantum yields of 1–2% were determined in aqueous solutions. The emission lifetimes indicate that no water molecules are present in the inner coordination sphere of Dy(III), which in the case of [Dy(CB-TE2PA)]^+^ was confirmed through the X-ray crystal structure. The ^1^H NMR paramagnetic shifts induced by Dy(III) were found to be dominated by the pseudocontact mechanism, though, for some protons, contact shifts are not negligible. The analysis of the pseudocontact shifts provided the magnetic susceptibility tensors of the three complexes, which were also investigated using CASSCF calculations. The transverse ^1^H relaxation data follow a good linear correlation with 1/*r*^6^, where *r* is the distance between the Dy(III) ion and the observed proton. This indicates that magnetic anisotropy is not significantly affecting the relaxation of ^1^H nuclei in the family of complexes investigated here.

## Abstract 2

Magnetic anisotropy has profound effects on the ^1^H NMR chemical shifts of a series of Dy(III) complexes but has a negligible impact on their relaxation rates.

## 1 Introduction

The lanthanide ions are a unique group of elements within the periodic table characterized by their similar chemical properties. In spite of being chemically very similar, the Ln(III) ions present very different optical and magnetic properties that are associated with their own specific electron configuration. Furthermore, the 4f orbitals are shielded from the environment by the external 5s^2^ and 5p^6^ electrons,^1^ and therefore do not significantly participate in the formation of chemical bonds.^2^ As a result, the coordination environment of the Ln(III) ion has a relatively minor impact on the optical and magnetic properties of the complex.

Some Ln(III) complexes form highly luminescent complexes that emit in the visible [i.e., Eu(III) and Tb(III)] or near-infrared regions [i.e., Pr(III), Nd(III), Ho(III), Er(III), or Yb(III)]. Concerning their magnetic properties, complexes of Gd(III) are widely used in clinical practice as contrast agents for magnetic resonance imaging. The [Xe]4f^7^ electron configuration of Gd(III) originates a symmetrical ^8^S electronic ground state, which makes this ion very efficient in promoting relaxation of active NMR nuclei in its vicinity. In MRI, the shortening of relaxation times of ^1^H water nuclei promoted by Gd(III) is used to generate contrast. Other paramagnetic Ln(III) ions induce relaxation rate enhancements in neighboring NMR nuclei, though the shorter electron relaxation times (∼10^–13^ s)^3^ result in longer *T*_1_ and *T*_2_ relaxation times. In contrast to Gd(III), all other Ln(III) ions form complexes affording observable NMR signals. Chemists have taken advantage of this feature since the early times of NMR, as paramagnetic lanthanide complexes with Ln(III) ions other than Gd(III) were found to induce significant paramagnetic chemical shifts without causing extensive line broadening. Thus, small Ln(III) complexes were routinely used as shift reagents to aid the analysis of NMR spectra of different substrates.^4^

The use of Ln(III) complexes as shift reagents has declined over the years as the increasing magnetic field strength of NMR spectrometers has provided enhanced spectral resolution. Nevertheless, Ln(III)-based paramagnetic tags are widely used for protein structure determination, as the paramagnetic shifts induced by the Ln(III) ions are dominated by the dipolar (pseudocontact) contribution,^5−7^ which encodes structural information. More recently, paramagnetic Ln(III) complexes were proposed as paraCEST^8−12^ and paraSHIFT imaging probes.^13−15^ The latter can be visualized directly in an MRI experiment, thanks to the paramagnetic effect that shifts ^1^H NMR signals of the probe well out of the region where endogenous signals are observed. In paraCEST probes, contrast is generated by the saturation of an NMR signal of protons exchanging with bulk water. The paramagnetism of the metal ion shifts this signal far from that of bulk water.^16^ From a more fundamental perspective, Ln(III)-induced paramagnetic chemical shifts and relaxation rate enhancement effects are widely used to obtain structural information in solution.^17−21^

The paramagnetic shifts induced by Ln(III) ions are reasonably well understood,^5,17,22^ though recent studies pointed out some limitations on the quantitative prediction of paramagnetic shifts across the series by Bleaney’s theory.^23−27^ The relaxation rates induced by Ln(III) ions other than Gd(III) are generally interpreted using the Bloch–Redfield–Wangsness theory,^28,29^ whose physical picture is based on the work by Solomon and Bloembergen.^30−32^ The theory relies on the point-dipole approximation and assumes that relaxation is isotropic. Recent studies reported unusual relaxation trends across the lanthanide series and suggested that the anisotropic contribution to relaxation may not be negligible, at least for nonsymmetrical systems.^33^

**Chart 1:**
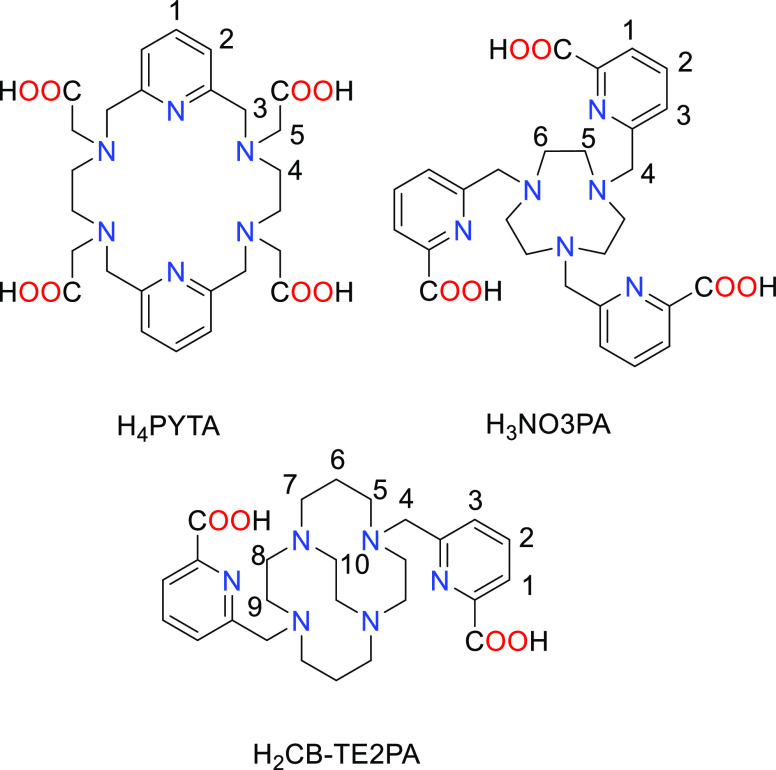
Structures of Ligands Discussed in the Present Work

In this work, we present a detailed analysis of the ^1^H NMR spectra of three Dy(III) complexes that provide different coordination environments. We have selected Dy(III) as a representative example of a Ln(III) ion that provides large pseudocontact shifts, actually the largest among the lanthanide series according to Bleaney’s theory,^34^ as well as strong relaxation enhancement effects.^35^ Furthermore, the splitting of the ^6^H_15/2_ ground state of Dy(III) can be analyzed using luminescence measurements, which can potentially provide rich electronic structure information. The three selected ligands form well-characterized Ln(III) complexes. The metal ion in the [Dy(PYTA)]^−^ complex is ten-coordinated by the ligand both in the solid state and in aqueous solution, with a *D*_2_ symmetry.^36^ The H_3_NO3PA ligand forms *C*_3_-symmetrical nine-coordinate complexes that were also characterized in the solid state and in solution.^37−39^ Finally, the Ln(III) complexes with H_2_CB-TE2PA^40,41^ display *C*_2_ symmetry and contain eight-coordinate metal ions for Ln = Eu–Lu (Chart 1).

In this work, the ^1^H NMR spectra of these complexes were measured and assigned in D_2_O solutions, which allowed determining their magnetic susceptibility tensors responsible for the pseudocontact shifts, as well as estimating the contact shift contributions to the different ^1^H NMR signals using density functional theory (DFT). A detailed photophysical study was also performed to gain information on the electronic structure of the complexes and the splitting of the ^6^H_15/2_ ground state. The ^1^H NMR spectra were subsequently measured at different magnetic fields (5.88, 7.05, 9.40, and 11.75 T). The *T*_2_ relaxation times obtained from line-width analysis were used to test the traditional relaxation theory,^29^ as expressed by the Solomon-Bloembergen equations for the dipolar relaxation and the equations describing the Curie-spin (CS) relaxation mechanism. We demonstrate that for this series of symmetrical complexes, relaxation is dominated by the isotropic contribution. The X-ray structure of the [Dy(CB-TE2PA)]^+^ complex is also reported.

## 2 Results and Discussion

### 2.1 X-ray Crystal Structure of [Dy(CB-TE2PA)](PF_6_)·2.5H_2_O

**Figure 1:**
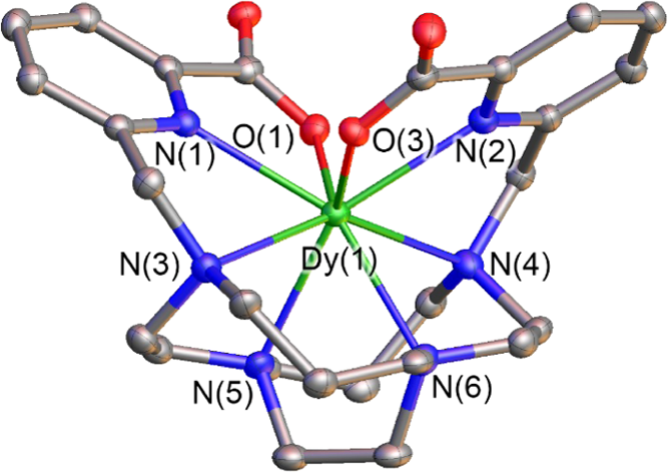
View of one of the [Dy(CB-TE2PA)]^+^ units present in crystals of [Dy(CB-TE2PA)](PF_6_)·2.5H_2_O with ellipsoids plotted at the 30% probability level. Hydrogen atoms, anions, and solvent molecules have been omitted for clarity. Selected bond distances (Å): Dy(1)-O(1), 2.276(6); Dy(1)-O(3), 2.309(5); Dy(1)-N(1), 2.469(7); Dy(1)-N(2), 2.464(7); Dy(1)-N(3), 2.613(7); Dy(1)-N(4), 2.598(7); Dy(1)-N(5), 2.539(7); Dy(1)-N(6), 2.537(7).

Crystals of the [Dy(CB-TE2PA)]^+^ complex suitable for X-ray diffraction studies were obtained from an aqueous solution of the complex containing an excess of KPF_6_. The asymmetric unit contains two slightly different [Dy(CB-TE2PA)]^+^ units, two PF_6_^–^ anions, and five water molecules that establish a hydrogen-bonding network with the carboxylate groups. A view of one of the [Dy(CB-TE2PA)]^+^ units, together with bond distances and angles, are provided in Figure 1.

The Dy(III) ion resides in the interior of the ligand cleft, being directly coordinated to the eight donor atoms of the ligand. The bond distances involving amine N atoms (∼2.53–2.62 Å) are in the low range observed for Dy(III) eight-coordinate complexes (2.54–2.68).^42−44^ The Dy–O bond distances are close to those reported for eight-coordinate complexes with polycarboxylate ligands.^42−44^ The two 1,4,7-triazacyclodecane units of CB-cyclam unit adopt irregular [2233] conformations,^45^ as observed previously for the La(III) and Eu(III) analogues. This is in contrast with the more common rectangular [2323] conformations observed for [In(CB-TE2PA)]^+^ and transition metal complexes with cross-bridge cyclam derivatives.^46−48^

### 2.2 Photophysical Properties

The magnetic anisotropy of Dy(III) complexes is related to the splitting of the ^6^H_15/2_ manifold caused by the ligand field.^49^ Absorption and emission spectroscopy can potentially provide detailed information on the electronic energy levels of Dy(III).^50^ Thus, we carried out a photophysical study to gain information on the electronic structure of the complexes investigated in this work.

**Figure 2:**
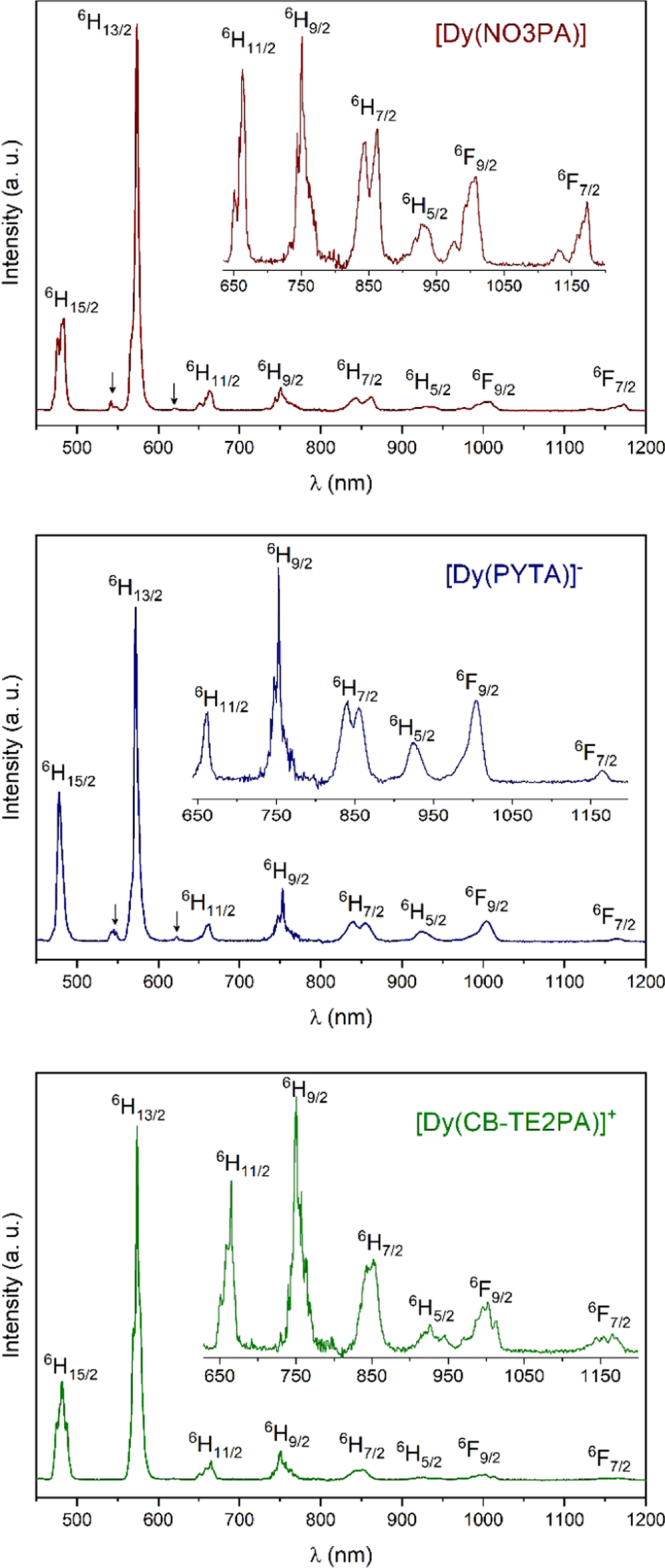
Emission spectra of the Dy(III) complexes recorded in D_2_O solutions showing the ^4^F_9/2_ → ^6^H_J_,^6^F_J_ transitions. The arrows identify ^4^I_15/2_ → ^6^H_J_ transitions. [Dy(NO3PA)], 1.03 × 10^–4^ M, pD = 7.4; [Dy(PYTA)]^−^, 1.02 × 10^–4^ M, pD = 7.2; [Dy(CB-TE2PA)]^+^, 1.02 × 10^–4^ M, pD = 7.1.

The absorption spectra of the [Dy(CB-TE2PA)]^+^ and [Dy(NO3PA)] complexes (D_2_O, pH 7.1–7.4) show a band with a maximum at ca. 275 nm characteristic of the picolinate chromophore (Figures S1 and S2, Supporting Information).^51,52^ The spectrum recorded for [Dy(PYTA)]^−^ displays an absorption band centered at 268 nm due to the pyridyl groups (Figure S3, Supporting Information). The emission spectra (Figure 2) recorded upon excitation through the ligand bands display the characteristic emission of Dy(III) in the visible region, with maxima around 480 (^4^F_9/2_ → ^6^H_15/2_ transitions), 575 (^4^F_9/2_ → ^6^H_13/2_), and 665 nm (^4^F_9/2_ → ^6^H_11/2_).^53^ The emission intensity in the visible region is largely dominated by the ^4^F_9/2_ → ^6^H_13/2_ transition at 575 nm (50–60%), as usually observed.^54−56^ Additionally, sizeable emission is also observed in the NIR region of the spectrum at ca. 850 (^4^F_9/2_ → ^6^H_7/2_), 930 (^4^F_9/2_ → ^6^H_5/2_), 1010 (^4^F_9/2_ → ^6^F_9/2_), and 1175 nm (^4^F_9/2_ → ^6^F_7/2_).^50^

The emission spectra recorded for the [Dy(NO3PA)] and [Dy(PYTA)]^−^ complexes display weak emission features that are shifted ∼1020 cm^–1^ to higher energies with respect to the ^4^F_9/2_ → ^6^H_J_ transitions (*J* = 15/2, 13/2 and 11/2). These weak emission features are attributed to transitions from the ^4^I_15/2_ level, which lies around 1020 cm^–1^ above the ^4^F_9/2_ level (Figure 2). The ^4^I_15/2_ → ^6^H_J_ transitions are, however, not observed for [Dy(CB-TE2PA)]^+^.

The three complexes investigated here display similar lifetimes in H_2_O solution, with values close to 20 μs (Table 1). These values are close to those determined in this solvent for Dy(III) complexes lacking water molecules in the inner coordination sphere (17–40 μs).^57−61^ Lifetimes are shorter in H_2_O than in D_2_O, as a result of the more efficient quenching effect of solvent O–H oscillators compared with O–D oscillators.^62,63^ The number of water molecules coordinated to the Dy(III) ion (*q*) can be estimated with an uncertainty of ±0.3 water molecules from these lifetimes using *q* = 24*k*_obs_ – 1.3, with *k*_obs_ = 1/τ_H_2_O_ (in μs^–1^).^64^ The values of *q* determined by this method (0.0 ± 0.3, Table 1) confirm the absence of coordinated water molecules in these complexes, in agreement with the corresponding X-ray crystal structures. Hydration numbers of Dy(III) complexes can be also estimated from lifetimes determined in H_2_O and D_2_O, using the relationship *q* = 2.61Δ*k*_obs_, with Δ*k*_obs_ = 1/τ_H_2_O_ – 1/τ_D_2_O_, in μs^–1^.^63^ The results again confirm the absence of water molecules in the inner coordination sphere (Table 1). However, the three complexes display significant differences in the lifetimes measured in D_2_O (Table 1), which range from ∼27 μs for [Dy(CB-TE2PA)]^+^ to ∼40 μs for [Dy(PYTA)]^−^.

**Table 1:** Main Spectroscopic Properties of the Dy Complexes in Water and Deuterated Water^a^

	λ_max_/nm	ε_D_2_O_/M^–1^ cm^–1^	ϕ_H_2_O_/%^a^^,^^b^	ϕ_D_2_O_/%^a^^,^^b^	τ_H_2_O_/μs^c^	τ_D_2_O_/μs^c^	*q*^d^	*q*^e^
[Dy(NO3PA)]	275	8820	2	4	20	30	–0.1	0.0
[Dy(PYTA)]^−^	268	5450	1	2	21	40	–0.2	0.1
[Dy(CB-TE2PA)]^+^	277	6850	1	2	18	27	0.0	0.1

a Measured in H_2_O or D_2_O pH (pD) = 7.2 (7.4), 7.2 (7.2), and 7.2 (7.1), respectively for [Dy(NO3PA)], [Dy(PYTA)]^−^, and [Dy(CB-TE2PA)]^+^.

b Calculated using Rhodamine 6G in water (ϕ_H_2_O_ = 0.76),^67^ estimated error ± 15%.

c Estimated error ± 10%.

d Calculated according to ref (64).

e Calculated according to ref (63).

Considering the relative weakness of the NIR emission bands, they were neglected in the calculation of the luminescence quantum yields (QYs), which were determined using the visible part only. The overall QYs measured in aqueous solutions are in the range 1–2% and roughly double in D_2_O. These values are similar to those determined for Dy(III) complexes lacking coordinated water molecules (0.1–3% in H_2_O),^57−61,65^ with the exception of a complex with bis-tetrazolate-pyridine ligand, which shows a considerably higher ϕ_H_2_O_ value of 7.1%.^66^

**Figure 3:**
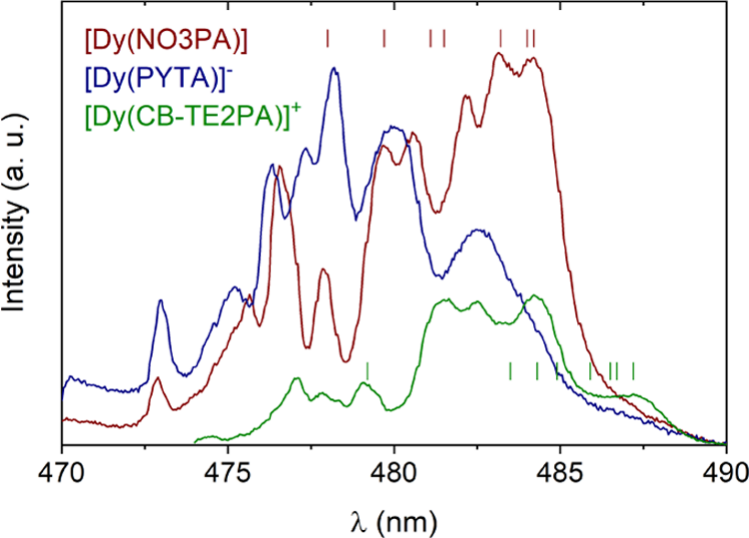
Emission spectra of the Dy(III) complexes recorded in frozen D_2_O solutions containing 50% glycerol (^4^F_9/2_ → ^6^H_15/2_ transitions). The small vertical lines show the splitting of the ^6^H_15/2_ manifold obtained with CASSCF/QDPT calculations.

Figure 3 presents a comparison of the high-resolution emission spectra obtained for the three complexes at 77 K in the region of the ^4^F_9/2_ → ^6^H_15/2_ transition (Figure 3). The spectra recorded for the three complexes show remarkable differences in the number and energy of the different components, as well as in the overall shape of the spectrum. This indicates that the crystal field splitting of the ^6^H_15/2_ and ^4^F_9/2_ levels is significantly different in these Dy(III) complexes. The emission spectrum recorded for [Dy(PYTA)]^−^ shows nine components that can be clearly identified, while the ^6^H_15/2_ multiplet splits into eight Kramers doublets by the effect of the crystal field. This suggests the emission spectrum contains contributions from hot emission bands, due to emission from different Kramers doublets of the ^4^F_9/2_ multiplet. This situation is even more obvious in the spectrum of [Dy(NO3PA)], which shows at least nine components. The thermal energy at 77 K is ∼53 cm^–1^, and thus different Kramers doublets of the excited ^4^F_9/2_ manifold may have significant populations even at this temperature. Measurements at a lower temperature (4 K) would be required for a more detailed analysis. An alternative reason for the presence of hot emission bands is that they arise from the population of excited Kramers doublets of the ^4^F_9/2_ manifold from deactivation of the ^4^I_15/2_ manifold. This appears to be reasonable, since weak emission peaks due to ^4^I_15/2_ → ^6^H_J_ transitions are observed in the emission spectra.

Theoretical CASSCF calculations reported previously for [Dy(NO3PA)] predicted an overall splitting of the ^6^H_15/2_ multiplet <300 cm^–1^ for the equilibrium geometry, though relatively small structural changes impacted significantly the energy of the Kramers doublets.^68^ Our calculations performed at the CASSCF/QDPT level (see Computational Details section below) provide a similar result, with an overall splitting of 266 cm^–1^ (Figure S4, Supporting Information). The splitting of the groups of Kramers doublets is also similar to that reported by Parker.^68^ The energies of the eight Kramers doublets are shown in Figure 3, taking as a reference the component of the emission spectrum with the lowest energy at 484.2 nm. The emission spectrum displays several components on the high-energy side, out of the range marked by the splitting of the ^6^H_15/2_ level. This again suggests that hot emission bands provide a significant contribution to the overall emission spectrum. A similar conclusion was achieved previously from the analysis of the emission spectra of Dy(III)^69^ and Yb(III)^70^ complexes. In the case of [Dy(CB-TE2PA)]^+^, the splitting of the ^6^H_15/2_ multiplet obtained with CASSCF/QDPT calculations also suggests that two components on the high-energy side arise from hot transitions. For [Dy(PYTA)]^−^, the emission spectrum shows a broad feature on the low energy side, which makes it difficult to locate the position of the Kramers doublet of the ^6^H_15/2_ multiplet with the lowest energy. Nevertheless, CASSCF/QDPT calculations provide an overall splitting of 251 cm^–1^ for this complex, which again suggests that hot emission bands (arising from thermally populated Kramers doublets of the ^4^F_9/2_ multiplet) are present in the high-energy side of the spectrum. Nevertheless, the emission spectra indicate that the different coordination numbers and coordination polyhedra of the three complexes have an important impact on the crystal field splitting of the ^6^H_15/2_ multiplet. Noteworthy, a large splitting of the ^6^H_15/2_ manifold of about 500 cm^–1^ was observed for [Dy(DOTA)]^−^,^71^ which indicates that the splitting of the Kramers doublets is rather sensitive to variations of the coordination environment.

### 2.3 ^1^H NMR Spectra

**Figure 4:**
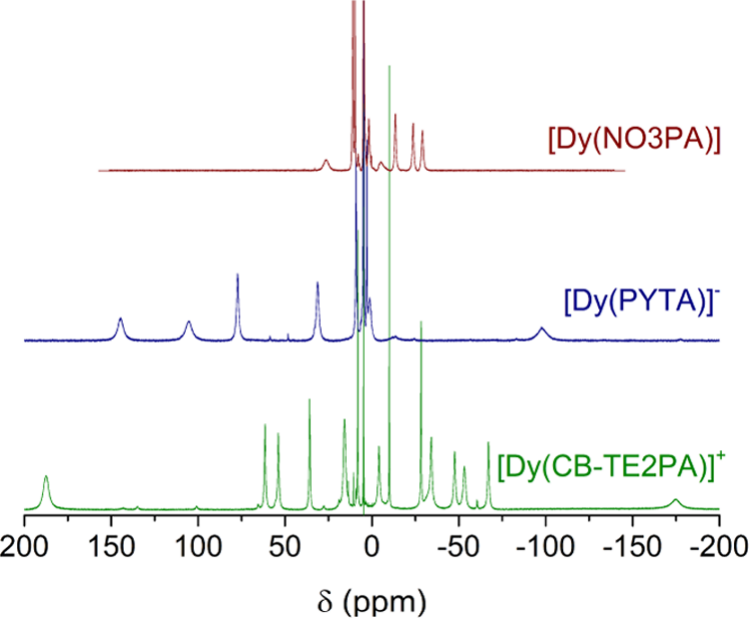
^1^H NMR spectra of the Dy(III) complexes investigated in this work (D_2_O, 298 K, 300 MHz, pH ∼ 7.0).

The ^1^H NMR spectra of the three complexes were recorded in D_2_O solution at pH ∼ 7.0 (Figure 4). The ^1^H NMR spectrum of the axially symmetrical [Dy(NO3PA)] complex displays the nine signals expected for an effective *C*_3_ symmetry in the chemical shift range +26 to −29 ppm (at 298 K). The spectrum was partially assigned previously by Parker et al.^68^ The full attribution of the spectrum was achieved using line-width analysis (Table 2), which allows identifying the axial and equatorial protons. Axial protons are generally closer to the paramagnetic center and thus provide broader signals.^35^ It is worth noting that the H5ax and H5eq protons of [Dy(NO3PA)] (Chart 1) display very similar Dy···H distances, contrary to what is observed for cyclen-based complexes. The metal ion in [Dy(NO3PA)] is placed well above the macrocycle plane (∼2.05 Å according to DFT calculations, see below), as a result of the small macrocyclic cavity. In cyclen-based complexes, the lanthanide resides only ∼1.65 Å above the macrocycle mean plane,^72^ resulting in short Dy···H_ax_ distances.

**Table 2:** ^1^H NMR Shifts (D_2_O, 288 K, pH 7.0, 400 MHz), Paramagnetic ^1^H NMR Shifts (δ^para^), and Geometric Factors for the [Dy(NO3PA)] Complex^a^

	H1	H2	H3	H4ax	H4eq	H5ax	H5eq	H6ax	H6eq
δ^obs^	9.86	10.99	11.37	28.67	1.73	–32.28	–15.05	–6.45	–25.60
δ^para^	2.10	2.79	3.37	24.65	2.41	–35.83	–17.67	–8.66	–28.51
(3 cos^2^ θ – 1)/*r*^3^/10^3^ Å^–3^^b^	–3.6543	–3.7431	–5.1772	–19.743	–2.4662	24.165	0.7444	–2.3630	9.9836

a See Chart 1 for labeling. Diamagnetic contributions taken from the shifts of the Lu(III) analogue, ref (38).

b Geometric factors obtained with DFT calculations (see computational methods).

The spectrum of [Dy(PYTA)]^−^ presents eight signals in the range +130 to −90 ppm at 298 K, which confirms an effective *D*_2_ symmetry of the complex in solution. A second set of paramagnetically shifted signals corresponding to a minor species present in solution is also observed. This minor species was attributed to a complex with a nine-coordinated metal ion in which one of the carboxylates remains uncoordinated.^36^ The integration of the ^1^H NMR signals indicates that the abundance of the minor species is ca. 10%. Finally, the [Dy(CB-TE2PA)]^+^ complex shows 17 signals in the ^1^H NMR spectrum, in line with an effective *C*_2_ symmetry in solution. These resonances are observed in the chemical shift range +190 to −175 ppm.

All three complexes investigated here adopt chiral point groups, and thus they exist in solution as racemic mixtures. For [Dy(NO3PA)], racemization requires a change in the rotation of the pendant arms and the inversion of the macrocyclic ring, as discussed for [Ln(DOTA)]^−^ derivatives.^73,74^ However, we did not observe any spectral changes that could be associated with the dynamics of the racemization process, even in the ^1^H NMR spectra recorded at high temperatures (see below). For [Dy(PYTA)]^−^ and [Dy(CB-TE2PA)]^+^, racemization requires full detachment of the acetate or picolinate pendant arms, and thus they are not likely to affect the ^1^H NMR spectra.

The paramagnetic chemical shifts of Dy(III) complexes are generally dominated by the pseudocontact contribution, which can be expressed as^5^
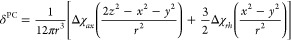
1Equation 1 assumes that the reference frame coincides with the orientation of the magnetic susceptibility tensor, whose axial and rhombic contributions are given by Δχ_ax_ and Δχ_rh_, respectively. Furthermore, *x*, *y* and *z* represent the Cartesian coordinates of a nucleus *i* relative to the location of the paramagnetic metal ion [Dy(III)] placed at the origin; and *r*^2^ = *x*^2^ + *y*^2^ + *z*^2^. In eq 1, the axial and rhombic parts of the magnetic susceptibility tensor as defined as
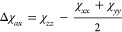
2
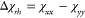
3The three complexes investigated here present comparable Dy(III)···H distances, as demonstrated by the DFT calculations presented below. However, the chemical shift range of these complexes increases as the symmetry of the complex decreases, which probably reflects an increased anisotropy of the magnetic susceptibility. Alternatively, significantly different contact contributions may be responsible for the different paramagnetic shifts observed for the three complexes.

The [Dy(NO3PA)] complex is axially symmetrical, as it possesses a symmetry axis *C_n_* with *n* ≥ 3. Under these circumstances, the rhombic term in eq 1 vanishes,^75^ which simplifies the analysis of the paramagnetic shifts. For axial symmetry, eq 1 can be rewritten in polar coordinates as follows:^5^
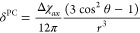
4Thus, a plot of the pseudocontact shifts versus the geometric term (3 cos^2^ θ – 1)/*r*^3^ should give a straight line passing through the origin with slope Δχ_ax_/12π. A plot of the observed paramagnetic shifts versus the geometric factors obtained using DFT shows a reasonable linear correlation (Figure S5, Supporting Information), affording a small Δχ_ax_ value (Table 3). This value is in very good agreement with that reported by Parker using ^1^H NMR data recorded in D_2_O (−5.3 × 10^–32^ m^3^).^27^

**Table 3:** Magnetic Susceptibility Tensors Determined from the Analysis of the Paramagnetic Shifts of Dy(III) Complexes (288 K)^a^

	NO3PA^3–^	PYTA^4–^	CB-TE2PA^2–^
Δχ_ax_/× 10^–32^ m^3^	–5.5 ± 1.0	18.7 ± 0.3	–8.1 ± 0.4
Δχ_rh_/× 10^–32^ m^3^		6.84 ± 0.6	21.4 ± 0.4

a The values for the complexes with PYTA^4–^ and CB-TE2PA^2–^ were obtained including contact shifts. The principal magnetic axes in [Dy(NO3A)] and [Dy(CB-TE2PA)]^+^ match the position of the *C*_3_ and *C*_2_ symmetry axes, respectively. For [Dy(PYTA)]^−^, the *z* axis bisects the ethylenediamine units, and the *x* axis contains the pyridyl N atoms.

The paramagnetic shifts observed for the [Dy(PYTA)]^−^ and [Dy(CB-TE2PA)]^+^ complexes were analyzed using eq 1, with Cartesian coordinates obtained using DFT calculations. A reasonable fit was obtained using this approach, which neglects contact contributions to the paramagnetic shifts. However, we noticed that the shifts calculated for some protons with eq 1 experience rather large deviations with respect to the observed chemical shifts, up to 22 ppm for [Dy(PYTA)]^−^ and 31 ppm for [Dy(CB-TE2PA)]^+^ (Table 4, see also Table S1, Supporting Information).

**Table 4:** ^1^H NMR Shifts (D_2_O, 288 K, pH 7.0, 400 MHz), Paramagnetic ^1^H NMR Shifts (δ^para^), Hyperfine Coupling Constants (*A*/ℏ), and Contact and Pseudocontact Contributions Calculated for the [Dy(CB-TE2PA)]^+^ Complex^a^

	δ^obs^	δ^para^	δ^para,calcd^	*A*/*ℏ*/10^6^ rad s^–1^^b^	δ^C^	δ^PC^	δ^PC,calcd^
H1	–30.93	–38.32	–31.34	–0.0028	–0.77	–37.55	–32.95
H2	–11.38	–18.81	–15.98	–0.0086	–2.41	–16.40	–16.17
H3	7.72	0.88	4.36	–0.0075	–2.09	2.97	5.88
H4ax	199.63	195.69	187.4	0.0072	2.00	193.7	197.6
H4eq	57.12	52.18	74.70	0.0759	–21.24	73.43	80.28
H5ax	205.3	201.7	205.9	–0.0100	–2.79	204.5	209.7
H5eq	65.48	62.48	90.63	–0.0575	–16.09	78.57	95.10
H6ax	12.31	9.88	12.67	0.0020	0.56	9.32	15.64
H6eq	38.66	37.06	36.19	0.0026	0.71	36.35	36.81
H7ax	–18.68	–22.21	–6.42	0.0240	6.74	–28.95	–20.24
H7eq	–37.58	–40.36	–31.37	0.0310	8.67	–49.03	–34.60
H8ax	–175.9	–179.99	–171.4	0.0460	12.88	–192.9	–184.2
H8eq	–52.56	–55.08	–65.05	0.0718	20.10	–75.18	–67.18
H9ax	11.04	7.16	10.35	–0.0100	–2.80	9.96	15.31
H9eq	–5.36	–8.49	–7.50	–0.0470	–13.16	4.67	10.58
H10ax	–65.40	–68.85	–56.58	–0.0053	–1.47	–67.38	–53.08
H10eq	–78.71	–81.27	–50.39	–0.0769	–21.52	–59.75	–48.93

a See Chart 1 for labeling. Diamagnetic contributions estimated from the ^1^H NMR spectrum of the La(III) analogue, ref (40).

b Hyperfine coupling constants calculated for [Gd(CB-TE2PA)]^+^ at the TPSSh/DKH2/DKH-def2-TZVPP level (see computational methods).

The contact NMR shifts induced by paramagnetic Ln(III) ions are related to a through-bond delocalization of unpaired spin density of the metal ion to the observed nucleus. The spin density at the observed nucleus is given by the scalar hyperfine coupling constant *A*/*ℏ*, while the contact shift δ^C^ can be approximated by eq 2,^17,76^ where ⟨*S*_*z*_⟩ is the spin expectation value of the lanthanide ion,^35^ γ_I_ is the nuclear gyromagnetic ratio, *k* is the Boltzmann constant, and β is the Bohr magneton.
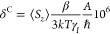
5Contact shifts are generally negligible for ^1^H nuclei placed five or more bonds away from the metal center but can be significant for nuclei close to the paramagnetic ion in terms of the number of bonds.^34,77^ In the particular case of Dy(III), pseudocontact shifts are expected to be roughly proportional to Bleaney’s constant *C_j_* = −100 determined for this ion, while the contact contribution is proportional to ⟨*S*_*z*_⟩ (28.565 for Dy(III)).^17^ Contact contributions are considered to be negligible for complexes of Yb(III), where *C*_*j*_/⟨*S*_*z*_⟩ = 8.5, but significant for Dy(III) (*C*_*j*_/⟨*S*_*z*_⟩ = −3.5).^21^

An estimate of the contact shifts was obtained by performing DFT calculations for the Gd(III) analogues, which provide straightforward access to the values of *A*/*ℏ*.^25,78,79^ The results of these calculations for [Dy(CB-TE2PA)]^+^ are presented in Table 4, while those obtained for [Dy(PYTA)]^−^ are presented in the Supporting Information (Table S1). Contact shifts were subsequently obtained from the *A*/*ℏ* values using eq 2 and ⟨*S*_*z*_⟩ = 22.0.^25^ The results evidence that contact contributions are small for axial protons of CH_2_ groups but sizeable for equatorial protons, representing up to ∼−21 ppm for H4eq in [Dy(PYTA)]^−^, and H4eq and H10eq in [Dy(CB-TE2PA)]^+^. The different contact contributions observed for axial and equatorial protons reflect the Karplus-like variation of the hyperfine coupling constant with the dihedral H-C-N-Dy angle.^78^

Once contact shifts were estimated, we analyzed the pseudocontact shifts using eq 1. The agreement between experimental and calculated shifts is very good, improving considerably with respect to the analysis neglecting contact shifts, as demonstrated by the agreement factor *AF*_*j*_, defined as^80^
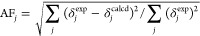
6Here, the sum runs over the different proton signals observed for a given complex. The analysis performed by neglecting contact contributions affords *AF*_*j*_ values of 0.149 and 0.169 for [Dy(CB-TE2PA)]^+^ and [Dy(PYTA)]^−^, respectively, which decrease significantly to 0.091 and 0.098 upon considering the contact shifts. Similar agreement factors have been obtained for Dy(III) complexes and are considered to be satisfactory.^77^ These results confirm that our DFT calculations provide at least rough estimates of the contact contributions and that the geometry of the complex obtained by DFT provides a reasonable approximation to the actual structure. Of note, the theoretical calculation of contact shifts for Ln(III) complexes with DFT is a difficult task.^81^ Furthermore, it has been recently demonstrated that dynamic effects may affect significantly the pseudocontact shifts; a recent study demonstrated that an idealized structure of [Dy(NO3PA)] provides an incomplete description of the system.^82^

The values of Δχ_ax_ and Δχ_rh_ obtained for [Dy(CB-TE2PA)]^+^ and [Dy(PYTA)]^−^ (Table 3) define rhombic magnetic susceptibility tensors, as would be expected, considering the symmetry of the complexes. In the case of [Dy(PYTA)]^−^, the axial contribution is still dominant, while for [Dy(CB-TE2PA)]^+^, the rhombic term is ca. 2.6 times larger than Δχ_ax_. The Δχ_ax_ value obtained for [Dy(NO3PA)] is about 5 times lower than those reported for Dy(III) DOTA derivatives,^83^ which highlights the small magnetic anisotropy of the former. The values Δχ_ax_ and Δχ_rh_ reported for Dy(III)-DOTA derivatives are, however, close to those obtained here for [Dy(CB-TE2PA)]^+^ and [Dy(PYTA)]^−^.^84^

### 2.4 ^1^H NMR Relaxation

Once the structures of the complexes in solution were established using paramagnetic ^1^H NMR spectroscopy, we envisaged to investigate ^1^H relaxation. The spectra presented above display rather broad ^1^H NMR signals, which makes the accurate determination of *T*_1_ relaxation times difficult. However, transverse relaxation times *T*_2_ can be obtained from the linewidths, which can be measured with good accuracy by fitting the experimental spectrum using Lorentzian–Gaussian functions. The paramagnetic contribution to the transverse relaxation rates receives contributions from the dipolar and Curie-spin mechanisms. The dipolar contribution *T*_2_^D^ is the result of a through-space interaction between the observed nucleus and the unpaired electron spins, originating from the fluctuating magnetic field associated with electron relaxation, eq 7:^17,30^
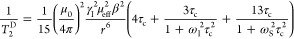
7Here, μ_0_/4π is the magnetic permeability of a vacuum, γ_I_ is the nuclear gyromagnetic ratio, μ_eff_ is the effective magnetic moment of the paramagnetic ion, β is the Bohr magneton, ω_I_ is the Larmor frequency of the nucleus, ω_S_ is the Larmor precession frequency for an electron, and *r* is the distance between the observed nuclei and the paramagnetic center. The correlation time τ_C_ depends on the rotational correlation time, τ_R_, and the electronic relaxation time *T*_1e_:
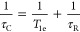
8The Curie-spin (CS) mechanism is also a dipolar effect arising from the interaction of the nuclear spin and the static magnetic moment of the electrons, associated with the difference in population of the electron spin levels due to the Boltzmann distribution:^85^
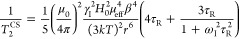
9Inspection of eq 9 evidences that the Curie-spin mechanism is expected to become more important as the applied magnetic field (*H*_0_) increases, particularly in the case of Ln(III) ions with high μ_eff_ values. Different authors have taken advantage of the dependence of the CS contribution with *H*_0_^2^ to estimate relative Ln···H distances, with plots of 1/*T_i_* (*i* = 1 or 2) versus *H*_0_^2^ generally showing good linear correlations.^35,36,86,87^Equations 7–9 were also used to analyze ^19^F relaxation rates in lanthanide complexes.^29^ It is important to mention that magnetic anisotropy may play a key role in the relaxation of ^1^H nuclei of small lanthanide complexes, with the anisotropic and isotropic parts providing similar contributions.^88^ Part of the motivation of the present work was to test the traditional relaxation theory (eqs 7–9) using a well-characterized set of complexes having different magnetic anisotropies. This is the case of the complexes investigated here, as demonstrated by the data reported in Table 3.

The linewidths of the ^1^H NMR signals of the complexes investigated here were obtained at four different magnetic fields (5.9, 7.05, 9.4, and 11.7 T) at 298 K. Furthermore, we also obtained a set of linewidths at 9.4 T and five different temperatures. We hypothesized that the variable field data would aid the fit of the relaxation data to eqs 7–9, allowing to discriminate the contributions of the dipolar and CS mechanisms. We have chosen a set of very rigid complexes, so that line broadening due to exchange effects provides negligible contributions to the linewidths. The measured linewidths are large (35–3600 Hz), and thus diamagnetic effects (∼4 Hz) were neglected.

**Figure 5:**
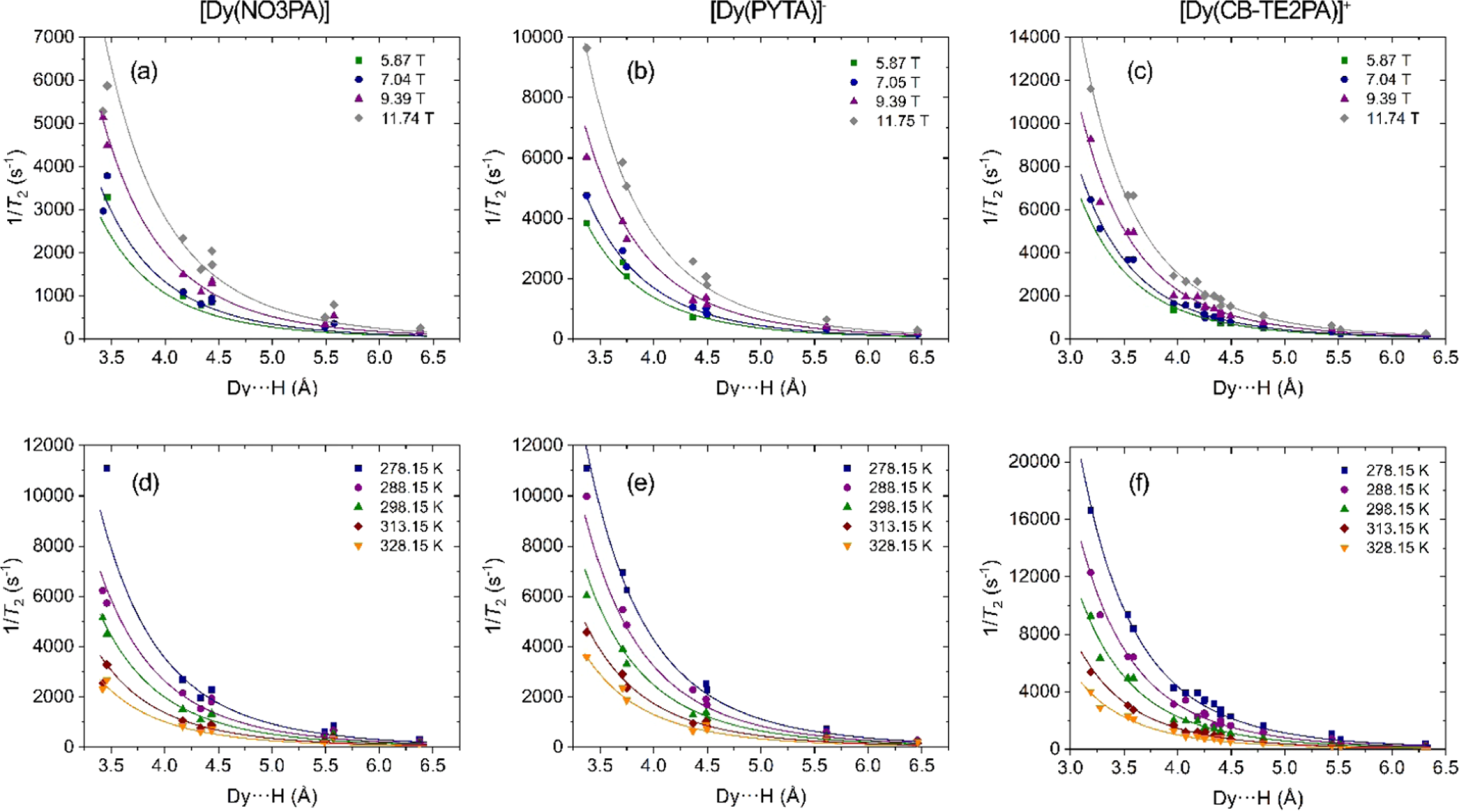
^1^H NMR transverse relaxation rates measured for Dy(III) complexes at different magnetic field strengths and temperatures (D_2_O, pH ∼ 7.0) versus the Dy···H distances obtained with DFT calculations. The solid lines represent the fits of the data, as explained in the text.

The relaxation rates obtained from line-width data display reasonably good linear correlations when plotted against 1/*r*^6^, with distances taken from DFT calculations (Figures S6–S8, Supporting Information). These results suggest that classical relaxation theory provides a reasonably good description of ^1^H relaxation in this family of complexes. We also note that *R*_2_ displays a linear dependence with the square of the applied magnetic field (Figures S9–S11, Supporting Information), as expected according to eq 6. The plots of 1/*T*_2_ versus the Dy···H distances also evidence that relaxation is faster upon increasing the magnetic field strength (Figure 5). The relaxation rates increase upon decreasing the temperature, which is mainly the combined effect of the 1/*T*^2^ dependence of the Curie-spin mechanism and the increased value of τ_R_ due to slow rotational motion at lower temperatures.

The transverse relaxation rates were fitted to eqs 7–9, assuming that both *T*_1e_ and τ_R_ display an Arrhenius dependence with temperature with activation energies *E*_R_ and *E*_V_, respectively.^89,90^ The fits are shown in Figure 5, while the fitted parameters are given in Table 5. Due to the relatively large number of parameters, we had to fix μ_eff_ to the common value of 10.64 BM.^91^ Furthermore, the value of *E*_v_ was fixed to 1 kJ mol^–1^ for [Dy(NO3PA)] and [Dy(PYTA)]^−^, as otherwise, small negative values were obtained. This has been observed previously very often when investigating the relaxation properties of Gd(III) complexes.^89,92,93^ Values of *E*_v_ close to 1 kJ mol^–1^ were reported for the lanthanide aqua ions.^3^

**Table 5:** Parameters Obtained from the Fits of Relaxation Data for Dy(III) Complexes^a^

	NO3PA^3–^	PYTA^4–^	CB-TE2PA^2–^
τ_R_/ps	122 ± 10	150 ± 6	122 ± 4
*T*_1e_/fs	196 ± 30	286 ± 39	375 ± 38
*E*_R_/kJ mol^–1^	21.3 ± 1.6	21.4 ± 0.9	26.2 ± 1.0
*E*_V_/kJ mol^–1^	1^a^	1^a^	11.9 ± 3.6

a Fixed during the fitting procedure.

The values of τ_R_ obtained from the fits of the data are very reasonable, considering the size of the complexes,^94^ with the slightly longer value of τ_R_ being observed for the complex with the highest molecular weight. One should note that the values of τ_R_ obtained from ^1^H NMRD studies on Gd(III) complexes are expected to be somewhat shorter due to the local mobility (internal motion) of the coordinated water molecule.^86^ The values of *E*_R_ are also close to those determined from ^1^H NMRD studies on Gd(III) complexes (∼20 kJ mol^–1^). For [Dy(CB-TE2PA)]^+^, the fit of the data afforded a rather high value of *E*_V_ (11.9 kJ mol^–1^), as well as a high value of *E*_R_. These values should be taken with some care, as these parameters are strongly correlated. It is, however, possible that the extreme rigidity of this complex results in a higher activation energy for the modulation of *T*_1e_.

The values of *T*_1e_ obtained for the three complexes are relatively similar, increasing from 196 fs for [Dy(NO3PA)] to 375 fs for [Dy(CB-TE2PA)]^+^. Very similar values were reported for the Dy(III) aqua-ion [Dy(H_2_O)_8_]^3+^ (299 fs)^3^ and for the [Dy(DOTA)]^−^ complex (∼250 fs).^35^ This suggests that *T*_1e_ is rather insensitive to the coordination environment around the metal ion. The short values of *T*_1e_ suggest that electron relaxation may originate from changes in the metal coordination environment caused by fast molecular vibrations.

## 3 Conclusions

The purpose of this work was to analyze ^1^H NMR relaxation in a series of well-characterized Dy(III) complexes. Luminescence measurements and the X-ray crystal structure of [Dy(CB-TE2PA)]^+^ were reported here for the sake of completeness. The selected complexes display different coordination numbers and thus coordination polyhedra, which results in markedly different magnetic anisotropies, which were obtained using ^1^H NMR measurements. The analysis of the transverse relaxation rates evidences that the traditional relaxation theory describes reasonably well the relaxation in these complexes, as evidenced by the linear dependence of 1/*T*_2_ with 1/*r*^6^. This indicates that anisotropic relaxation does not provide a significant contribution for the complexes investigated here. We note that the effect of anisotropy is expected to be small while being one order of magnitude smaller than the average susceptibility.^95^ This situation is observed for the studied complexes, as Δχ_ax_ and Δχ_rh_ represent <15% of χ_iso_ (∼151 × 10^–32^ m^3^). In the case of [Dy(NO3PA)], Δχ_ax_ is particularly small compared with χ_iso_ (<4%). However, we do not exclude that anisotropic relaxation may play a role in Ln(III) complexes with very large magnetic anisotropies.

## 4 Experimental and Computational Section

### 4.1 General

All solvents and reagents used were purchased from commercial sources and were used as supplied. Ligands H_3_NO3PA,^38^ H_2_CB-TE2PA,^40^ and H_4_PYTA^36^ were synthesized according to previously reported procedures. The Dy(III) complexes [Dy(PYTA)]^−^ and [Dy(NO3PA)] were prepared by mixing equimolar amounts of ligand and Dy(NO_3_)_3_·6H_2_O in either D_2_O or H_2_O and adjustment of the pH to ∼7.0 using a diluted NaOH solution. [Dy(CB-TE2PA)]^+^ was prepared in a microwave apparatus, following the procedure reported for the Eu(III) complex,^40^ and the resulting complex was then redissolved in either H_2_O or D_2_O. NMR measurements were recorded using complex concentrations of ∼30 mM. ^1^H NMR spectra were recorded in Bruker DPX 250 (5.87 T), Bruker Avance 300 (7.05 T), Bruker ARX400 (9.40 T), and Bruker Avance 500 (11.75 T) spectrometers. Linewidths were measured with the deconvolution tool of MestRe Nova,^96^ using Lorentzian–Gaussian functions (Figure S13, Supporting Information). Single crystals of [Dy(CB-TE2PA)](PF_6_)·2.5H_2_O were obtained by slow evaporation of an aqueous solution of the complex in the presence of excess KPF_6_.

### 4.2 Luminescence Measurements

Spectroscopic measurements were performed with 10 × 10 mm^2^ Quartz Suprasil certified cells (Hellma Analytics). UV/Vis absorption spectra were recorded on a Lambda 950 UV/VIS/NIR absorption spectrometer from PerkinElmer. Steady-state emission spectra were recorded on an Edinburgh Instruments FLP920 working with a continuous 450 W Xe lamp and a red sensitive R928 photomultiplier from Hamamatsu in Pelletier housing for visible detection (230 to 900 nm) or a Hamamatsu R5 509-72 photomultiplier cooled at 77 K for the Vis-NIR part. A 330 nm high pass cutoff filter was used to eliminate the second-order artifacts for the visible part, and an 850 nm high pass cutoff filter for the NIR part. Luminescence lifetimes were measured on the same instrument working in the multichannel spectroscopy mode and using a Xenon flash lamp as the excitation source. The decay curves were corrected for the intensity profile of the lamp by measuring the diffraction signals of a scattering sample of colloidal silica. Errors in the luminescence lifetimes are estimated to be ±10%. Luminescence quantum yields were measured according to conventional procedures, with diluted solutions (optical density < 0.05 at the excitation wavelength), using Rhodamine 6G in water (φ = 76.0%).^67^ High-resolution emission spectra were recorded at 77 K using a nitrogen-cooled Oxford Instruments cryostat with 0.05 nm slits at the emission, except for [Dy(CB-TE2PA)]^+^, for which slits of 0.1 nm were used due to the weaker intensity.

### 4.3 X-ray Diffraction Measurements

A crystal of [Dy(CB-TE2PA)](PF_6_)·2.5H_2_O was analyzed by X-ray diffraction. Crystallographic data and structure refinement parameters are shown in Table S2 (Supporting Information). Crystallographic data were collected on a Bruker D8 Venture diffractometer with a Photon 100 CMOS detector at 100 K with Mo Kα radiation (λ = 0.71073 Å) generated by an Incoatec high brilliance microfocus source equipped with Incoatec Helios multilayer optics. APEX3^97^ software was used for collecting frames of data, indexing reflections, and the determination of lattice parameters, while SAINT^98^ was used for the integration of the intensity of reflections and SADABS^99^ for scaling and empirical absorption correction. The structure was solved by dual-space methods using the program SHELXT.^100^ All non-hydrogen atoms were refined with anisotropic thermal parameters by full-matrix least-squares calculations on F^2^ using the program SHELXL-2014.^101^ Hydrogen atoms of the compound were inserted at calculated positions and constrained with isotropic thermal parameters. CCDC 2257994 contains supplementary crystallographic data, which can be obtained free of charge from the Cambridge Crystallographic Data Centre via www.ccdc.ac.uk/data_request/cif.

### 4.4 Computational Details

The geometries of the Dy(III) complexes were optimized using Gaussian 16,^102^ employing the hybrid-meta GGA functional TPSSh,^103^ in combination with the small-core quasi-relativistic effective core potential proposed by Dolg et al.^104^ (28 electrons, 1s-3d, in the core for Dy) and the associated ECP28MWB_GUESS basis set, which possesses a (42s26p20d8f)/[3s2p2d1f] contraction scheme. The standard Def2-TZVPP basis set was used for the ligand atoms.^105^ Solvent effects were incorporated using the polarized continuum model (IEF-PCM variant).^106,107^ Frequency calculations were employed to confirm that the optimized geometries corresponded to true energy minima.

Complete active space self-consistent field (CASSCF)^108^ calculations were carried out using the ORCA program package (version 5.0.3).^109,110^ The active space included the nine electrons of Dy(III) distributed over the seven 4f orbitals [CASSCF(9,7)]. The state average CASSCF calculation included 21 sextet, 224 quartet, and 490 doublet roots. In these calculations, we used the SARC2-DKH-QZVP^111^ basis set for Dy and its associated SARC2-DKH-QZVP/JK auxiliary basis set to accelerate the calculations with the resolution of identity and chain of spheres (RIJCOSX)^112,113^ method. For ligand atoms, we used the DKH-def2-TZVPP^105^ basis set and auxiliary basis sets generated by ORCA with the Autoaux^114^ procedure. Relativistic effects were taken into account with the Douglas–Kroll–Hess (DKH2) method^115,116^ using a finite nucleus model.^117^ SOC effects were incorporated using quasi-degenerate perturbation theory (QDPT).^118,119^ Solvent effects were included using the SMD solvation model.^120^

## Supporting Information Available

Absorption and emission spectra, additional NMR data, and computational data (PDF)
