# Supplementary material for: Effect of Magnetic Anisotropy on the 1H NMR Paramagnetic Shifts and Relaxation Rates of Small Dysprosium(III) Complexes
Source: Inorg Chem. 2023 Aug 21;62(35):14326–38. doi: 10.1021/acs.inorgchem.3c01959 (PMC10481378; doi:10.1021/acs.inorgchem.3c01959)
Supplement: Supplementary file 1 — ic3c01959_si_001.pdf [file ic3c01959_si_001.pdf]

Supporting Information for:

Effect of Magnetic Anisotropy on the ^{1}H NMR Paramagnetic Shifts and Relaxation Rates of Small Dysprosium(III) Complexes

Charlene Harriswangler,† Fátima Lucio-Martínez,† Léna Godec,‡ Lohona Kevin Soro,‡ Sandra Fernández-Fariña,‖ Laura Valencia,§ Aurora Rodríguez-Rodríguez,† David Esteban-Gómez,† Loïc J. Charbonnière,‡ and Carlos Platas Iglesias*,†

† Universidade da Coruña, Centro Interdisciplinar de Química e Bioloxía (CICA) and Departamento de Química, Facultade de Ciencias, 15071, A Coruña, Galicia, Spain

‡ Equipe de Synthèse Pour l'Analyse (SynPA), Institut Pluridisciplinaire Hubert Curien (IPHC), UMR 7178, CNRS, Université de Strasbourg, ECPM, 25 rue Becquerel, 67087, Strasbourg Cedex, France

§ Departamento de Química Inorgánica, Facultad de Ciencias, Universidade de Vigo, As Lagoas, Marcosende, 36310 Pontevedra, Spain

‖ Departamento de Química Inorgánica, Facultade de Química, Campus Vida, Universidade de Santiago de Compostela, 15782 Santiago de Compostela, Spain

Email: carlos.platas.iglesias@udc.es

Contents :

Figure S1: Absorption spectrum of $[Dy(CB\text{-}TE2PA)]^+$ (1.02 x 10^{-4} M in D_2O, pD = 7.1)....3

Figure S2:Absorption spectrum of [Dy(NO3PA)] (1.03 x 10^{-4} M in D_2O, pD = 7.4)....3

Figure S3: Absorption spectrum of $[Dy(PYTA)]^-$ (1.02 x 10^{-4} M in D_2O, pD = 7.2)....4

Figure S4: Energy levels of the $^6H_{15/2}$ manifold obtained with CASSCF/QDPT calculations....5

Figure S5: Linear fit of δ^{para} plotted against the geometric term $(3\cos^2\theta\text{-}1)/r^3$ for [Dy(NO3PA)]....6

Table S1: Paramagnetic ^{1}H NMR shifts (δ^{para}, D_2O, 288 K, pH 7.0, 400 MHz), hyperfine coupling constants ($A/\hbar$) and contact and pseudocontact contributions calculated for the $[Dy(PYTA)]^-$ complex....6

Figure S6: Relaxation rates plotted against $1/r^6$ at different magnetic field strengths (left) and temperatures (right) for [Dy(NO3PA)]....7

Figure S7: Relaxation rates plotted against $1/r^6$ at different magnetic field strengths (left) and temperatures (right) for $[Dy(PYTA)]^-$....7

Figure S8: Relaxation rates plotted against $1/r^6$ at different magnetic field strengths (left) and temperatures (right) for $[Dy(CB\text{-}TE2PA)]^+$....7

Figure S9: Relaxation rates of each proton plotted against B^2 for [Dy(NO3PA)]....8

Figure S10: Relaxation rates of each proton plotted against B^2 for $[Dy(PYTA)]^-$....8

Figure S11: Relaxation rates of each proton plotted against B^2 for $[Dy(CB\text{-}TE2PA)]^+$....9

Figure S12: Mass spectrum recorded from an aqueous solution of the $[Dy(CB\text{-}TE2PA)]^+$ complex (ESI+)....10

Figure S13: Mass spectrum recorded from an aqueous solution of the $[Dy(PYTA)]^-$ complex (ESI-)....11

Figure S14: Mass spectrum recorded from an aqueous solution of the [Dy(NO3PA)] complex (ESI+). 12

Figure S15. Fits of the ^{1}H NMR signals of Dy(PYTA)] to Lorentzian functions and linewidths. The diamagnetic region shows small signals due to a slight ligand excess (2.3-3.5 ppm) that have been deconvoluted. 13

Table S2: Crystal data and structure refinement for [Dy(CB-TE2PA)](PF_6)·2.5H_2O. 14

Table S3: Cartesian coordinates (Å) of the [Dy(NO3PA)] complex optimized with DFT. 14

Table S4: Cartesian coordinates (Å) of the $[Dy(PYTA)]^-$ complex optimized with DFT. 15

Table S5: Cartesian coordinates (Å) of the $[Dy(CB-TE2PA)]^+$ complex optimized with DFT. 17

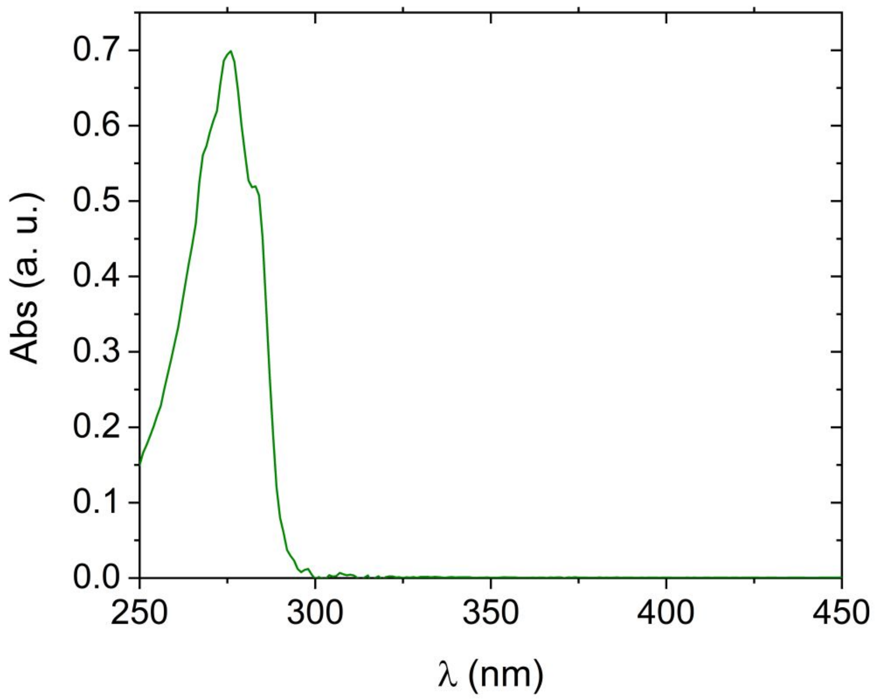

Figure S1: Absorption spectrum of $[Dy(CB\text{-}TE2PA)]^+$ (1.02 x 10^{-4} M in D_2O, pD = 7.1).

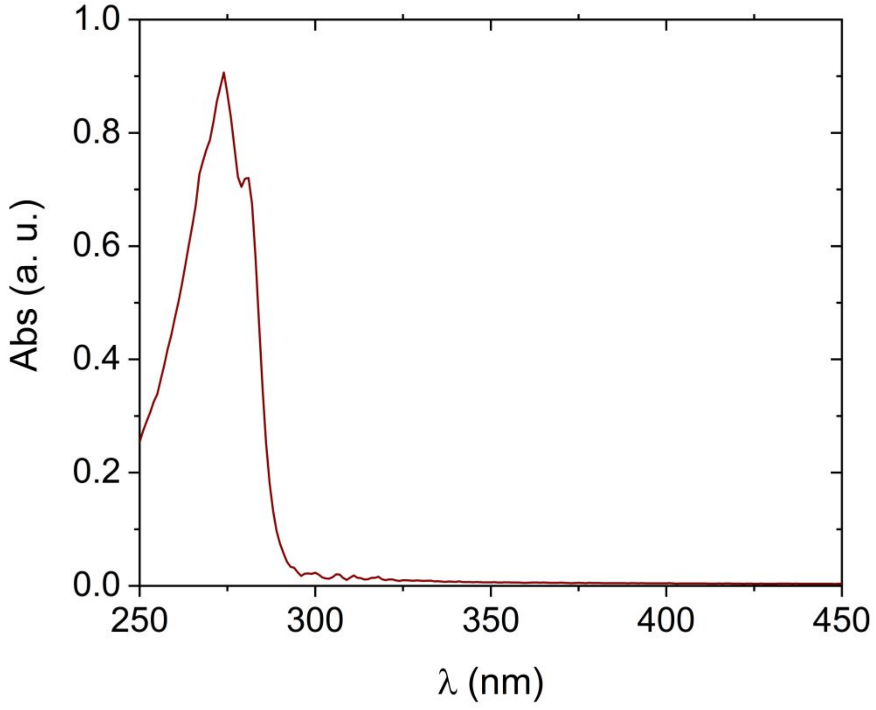

Figure S2:Absorption spectrum of [Dy(NO3PA)] (1.03 x 10^{-4} M in D_2O, pD = 7.4).

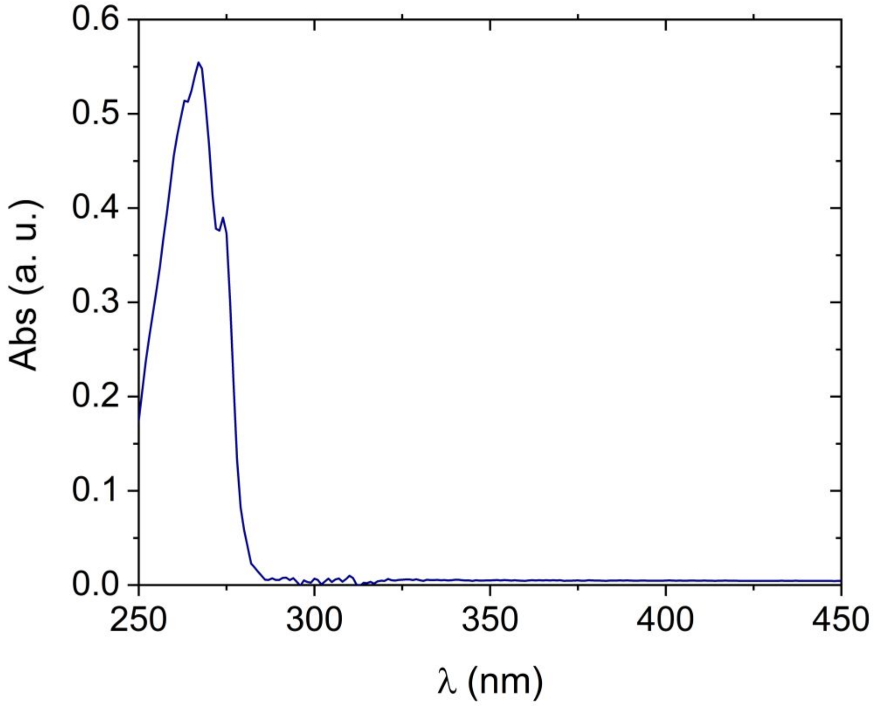

Figure S3: Absorption spectrum of $[Dy(PYTA)]^-$ (1.02 x 10^{-4} M in D_2O, pD = 7.2).

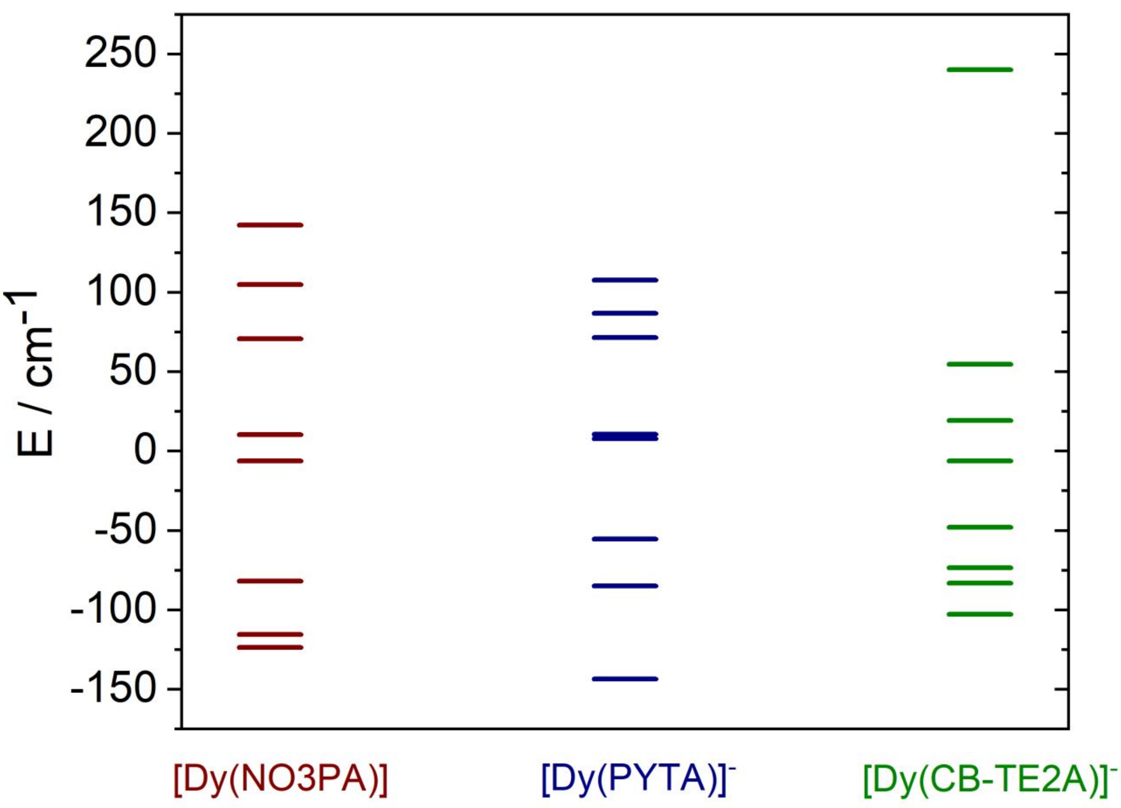

Figure S4: Energy levels of the $^6H_{15/2}$ manifold obtained with CASSCF/QDPT calculations.

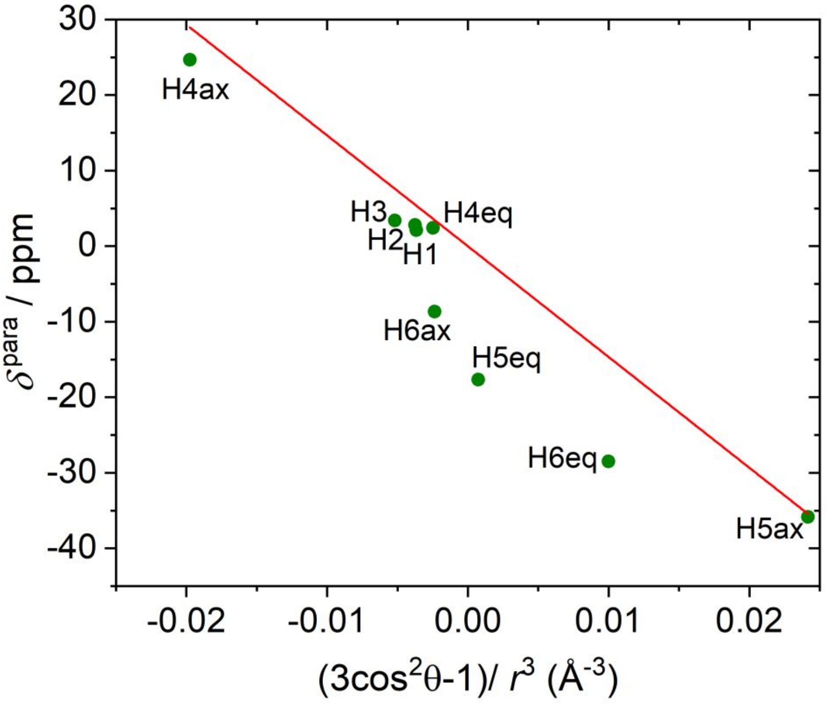

Figure S5: Linear fit of δ^{para} plotted against the geometric term $(3\cos^2\theta-1)/r^3$ for [Dy(NO3PA)].

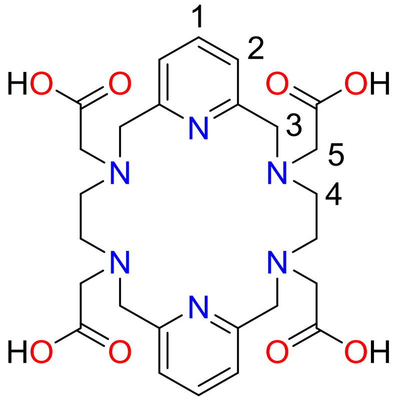

Table S1: Paramagnetic ^{1}H NMR shifts (δ^{para}, D_2O, 288 K, pH 7.0, 400 MHz), hyperfine coupling constants ($A/\hbar$) and contact and pseudocontact contributions calculated for the $[Dy(PYTA)]^-$ complex.

	H1	H2	H3ax	H3eq	H4ax	H4eq	H5ax	H5eq
δ^{obs}	-1.95	2.39	91.98	21.95	140.0	72.00	-102.3	-0.92
δ^{para}	-9.85	-5.02	88.19	17.46	137.4	68.82	-105.5	-4.47
$\delta^{para,calc}$	-7.66	-2.79	81.96	37.01	126.0	90.84	-96.91	5.68
$A/\hbar$ / 10^6 rad s^{-1} [b]	-0.0056	-0.0015	0.0088	-0.0464	-0.0018	-0.0774	0.0197	-0.0183
δ^{C}	-1.57	-0.42	2.46	-13.00	-0.50	-21.68	5.52	-5.13
δ^{PC}	-8.29	-4.60	85.72	30.46	137.9	90.50	-111.0	0.66
$\delta^{PC,calc}$	-8.30	-3.12	87.45	39.44	134.84	97.16	-93.85	6.19

[a] See Chart 1 for labelling. [b] Hyperfine coupling constants calculated for $[Gd(PYTA)]^-$ at the TPSSh/DKH2/DKH-def2-TZVPP level (see computational methods).

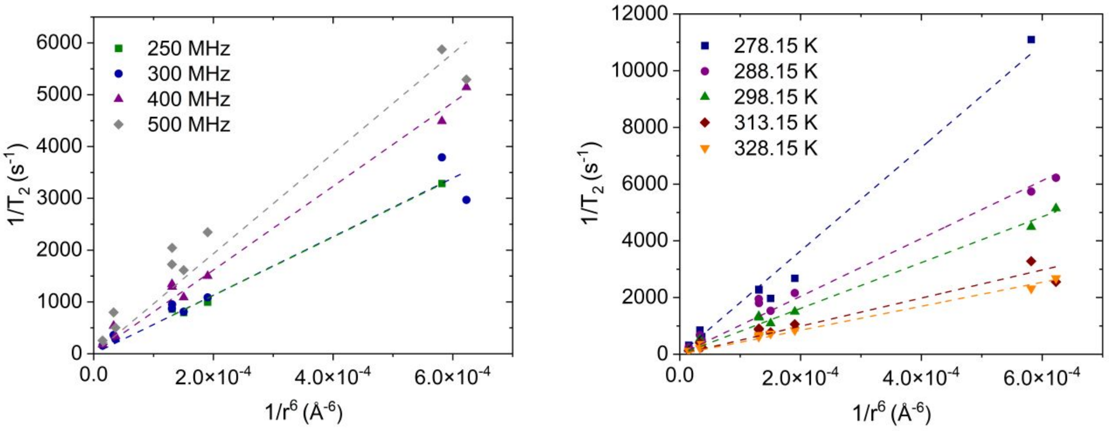

Figure S6: Relaxation rates plotted against $1/r^6$ at different magnetic field strengths (left) and temperatures (right) for [Dy(NO3PA)].

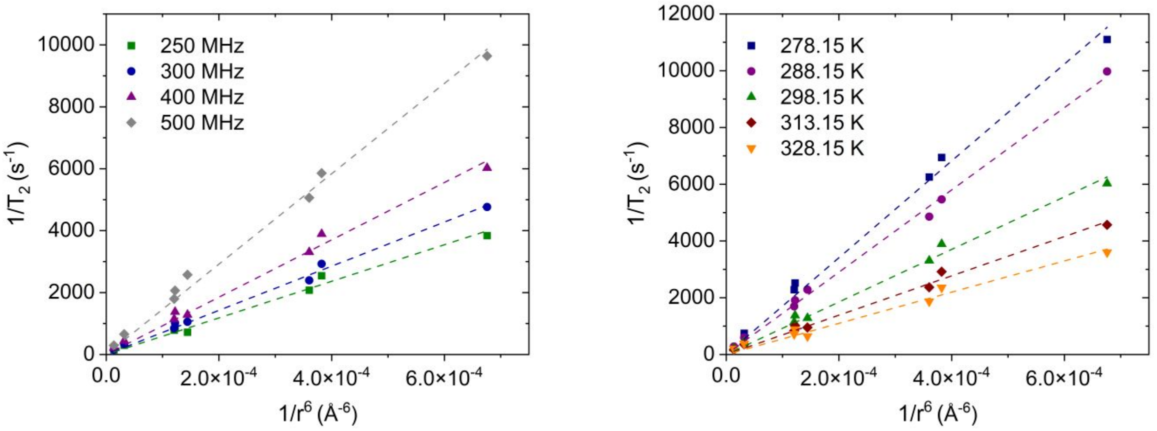

Figure S7: Relaxation rates plotted against $1/r^6$ at different magnetic field strengths (left) and temperatures (right) for $[Dy(PYTA)]^-$.

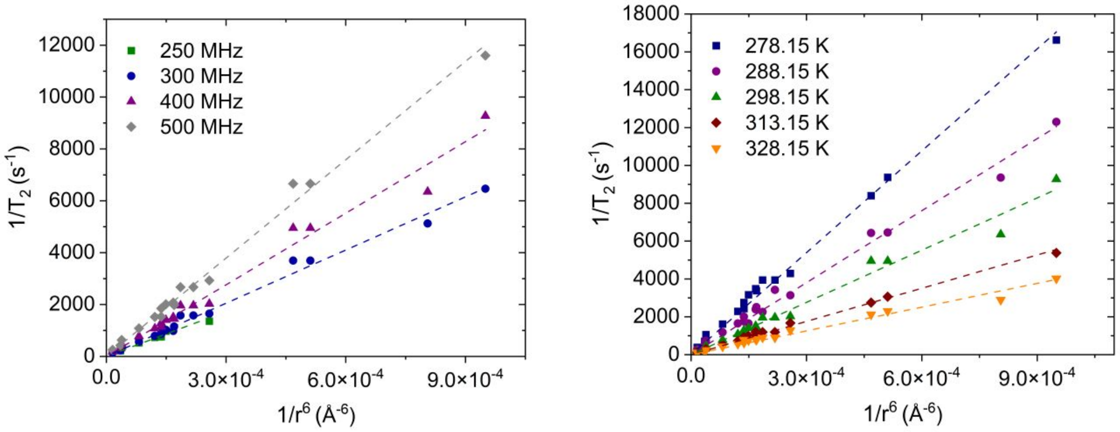

Figure S8: Relaxation rates plotted against $1/r^6$ at different magnetic field strengths (left) and temperatures (right) for $[Dy(CB\text{-}TE2PA)]^+$.

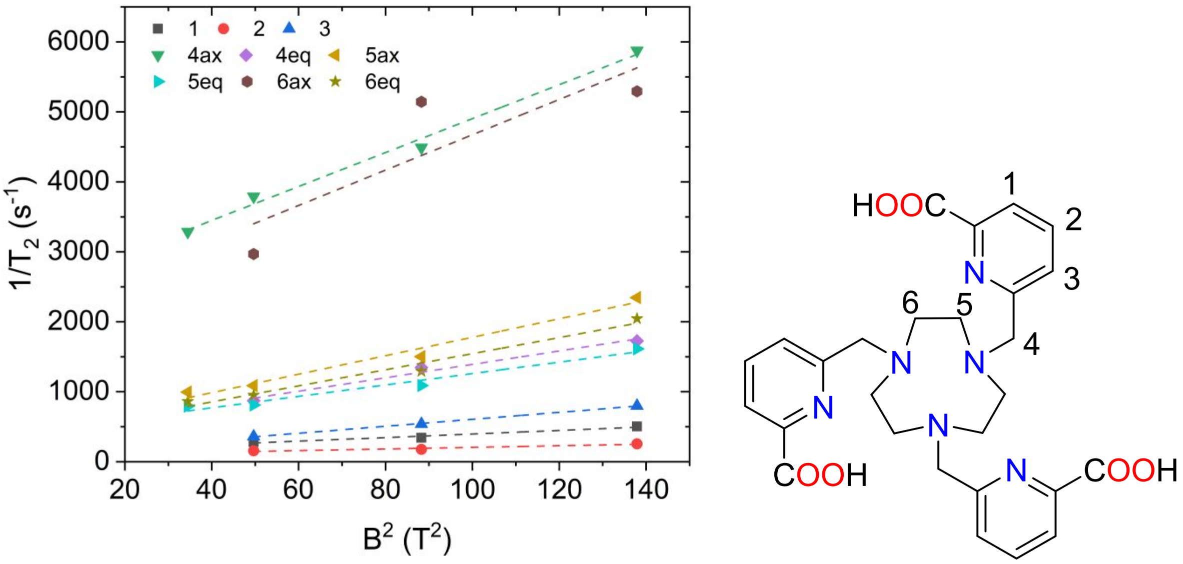

Figure S9: Relaxation rates of each proton plotted against B^2 for [Dy(NO3PA)].

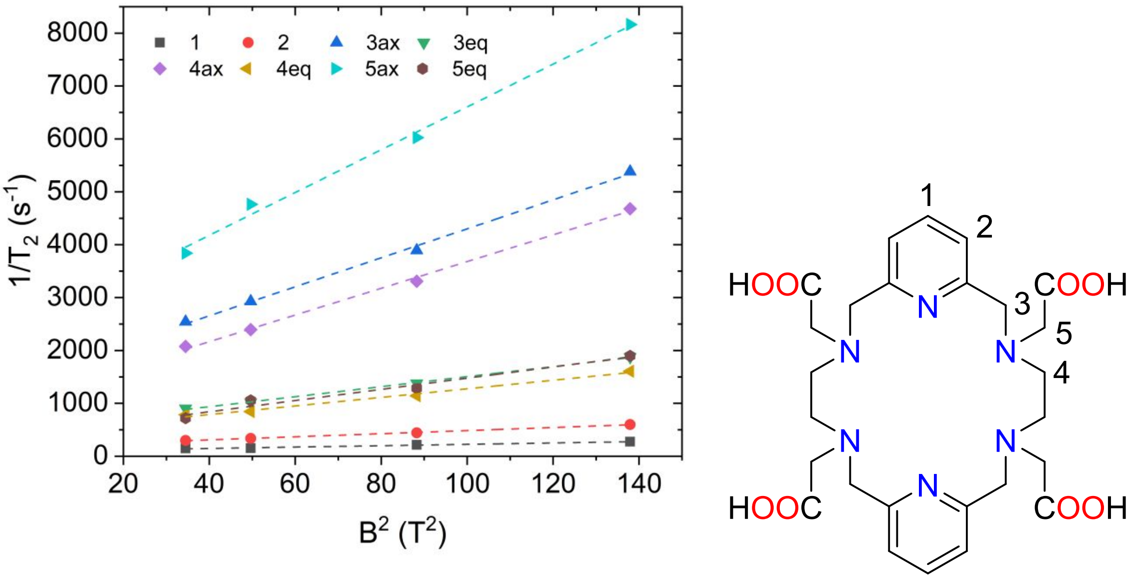

Figure S10: Relaxation rates of each proton plotted against B^2 for $[Dy(PYTA)]^-$

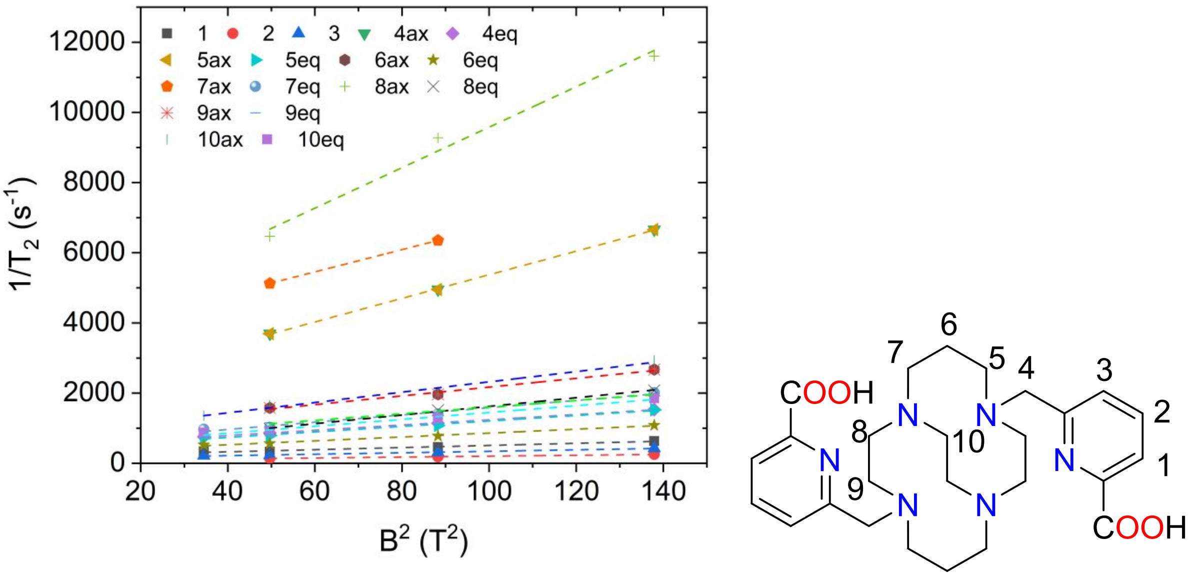

Figure S11: Relaxation rates of each proton plotted against B^2 for $[Dy(CB\text{-}TE2PA)]^+$

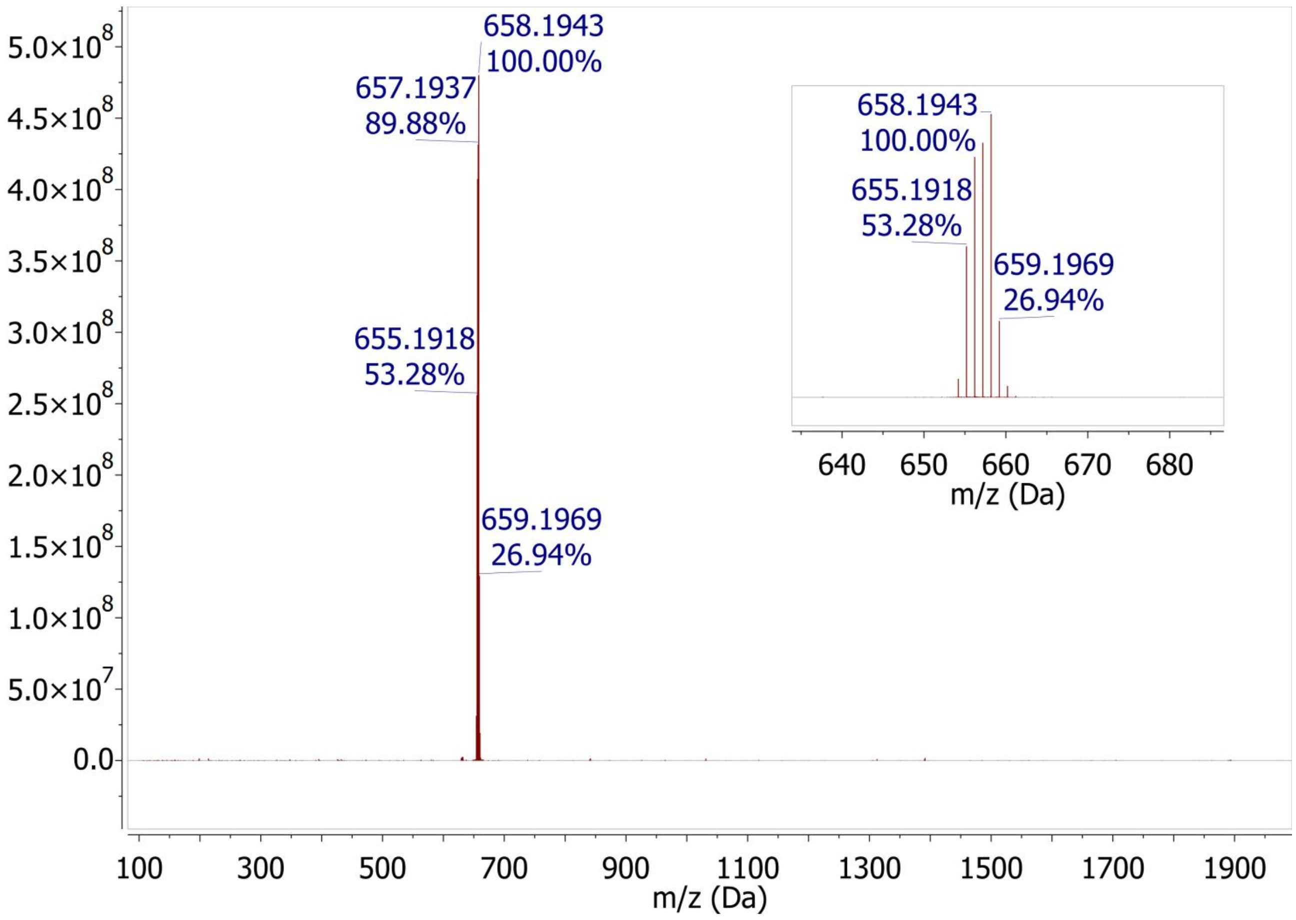

Figure S12: Mass spectrum recorded from an aqueous solution of the $[Dy(CB\text{-}TE2PA)]^{+}$ complex (ESI^{+}).

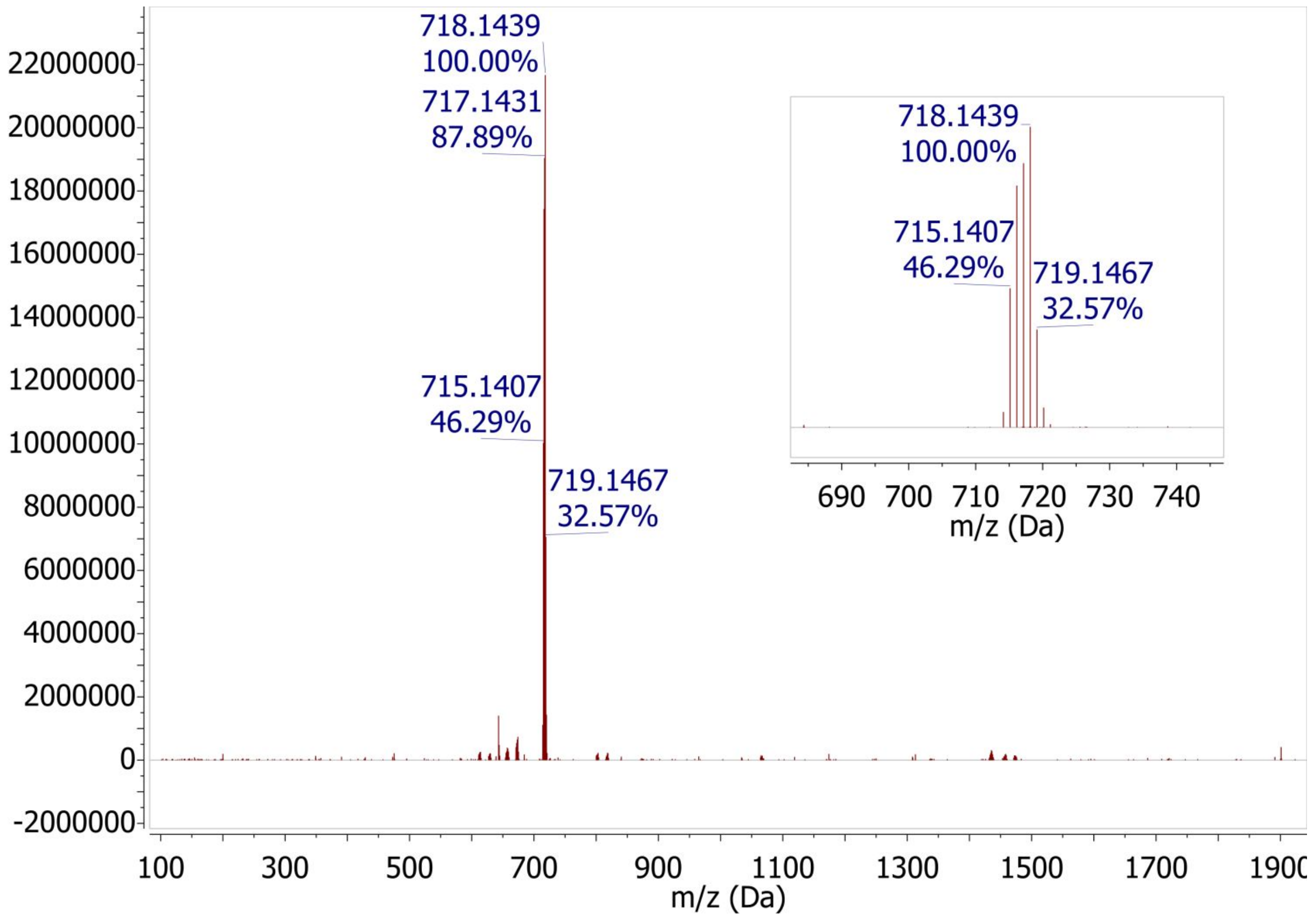

Figure S13: Mass spectrum recorded from an aqueous solution of the [Dy(PYTA)]$^{-}$ complex (ESI^{-}).

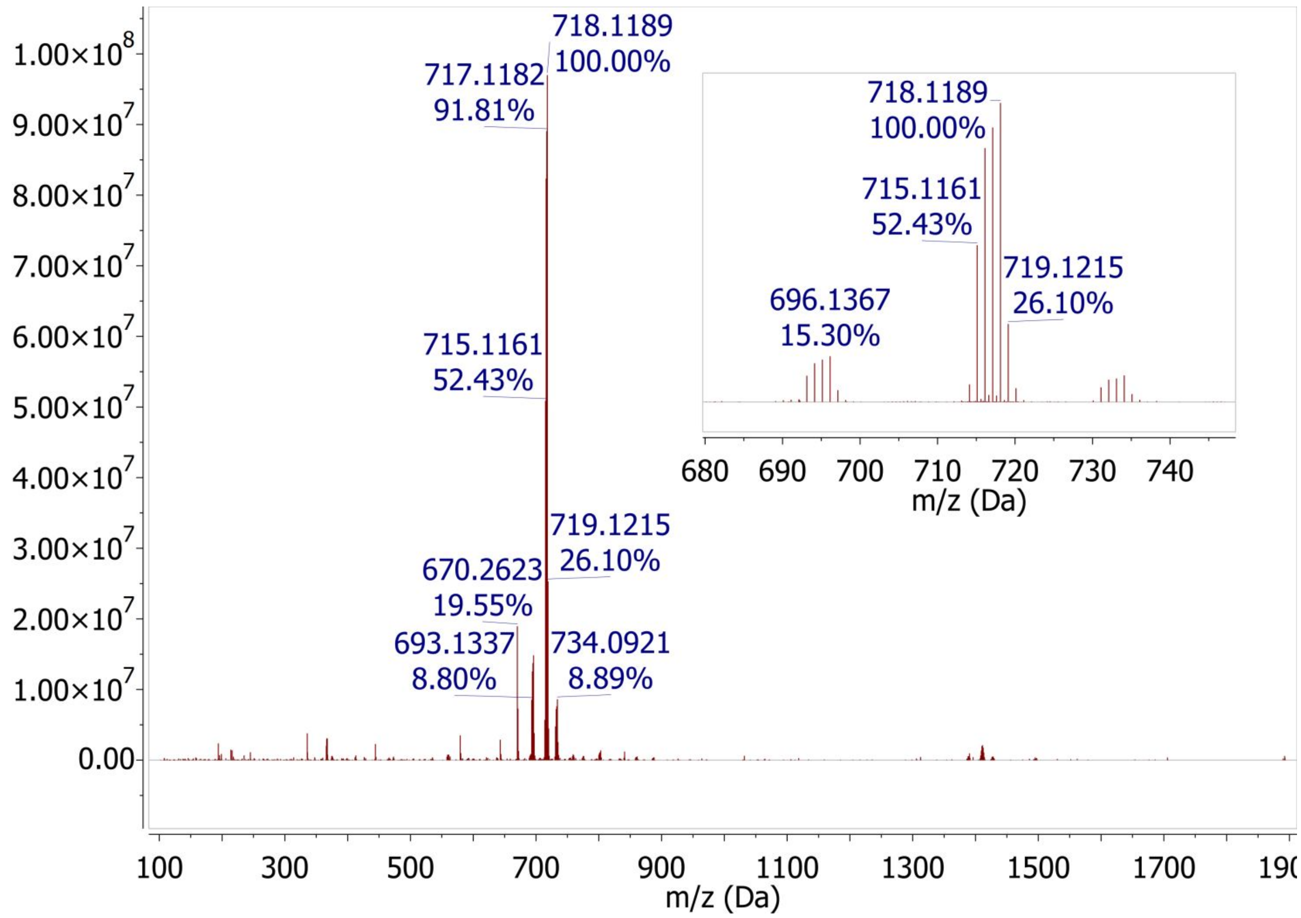

Figure S14: Mass spectrum recorded from an aqueous solution of the [Dy(NO3PA)] complex (ESI^+).

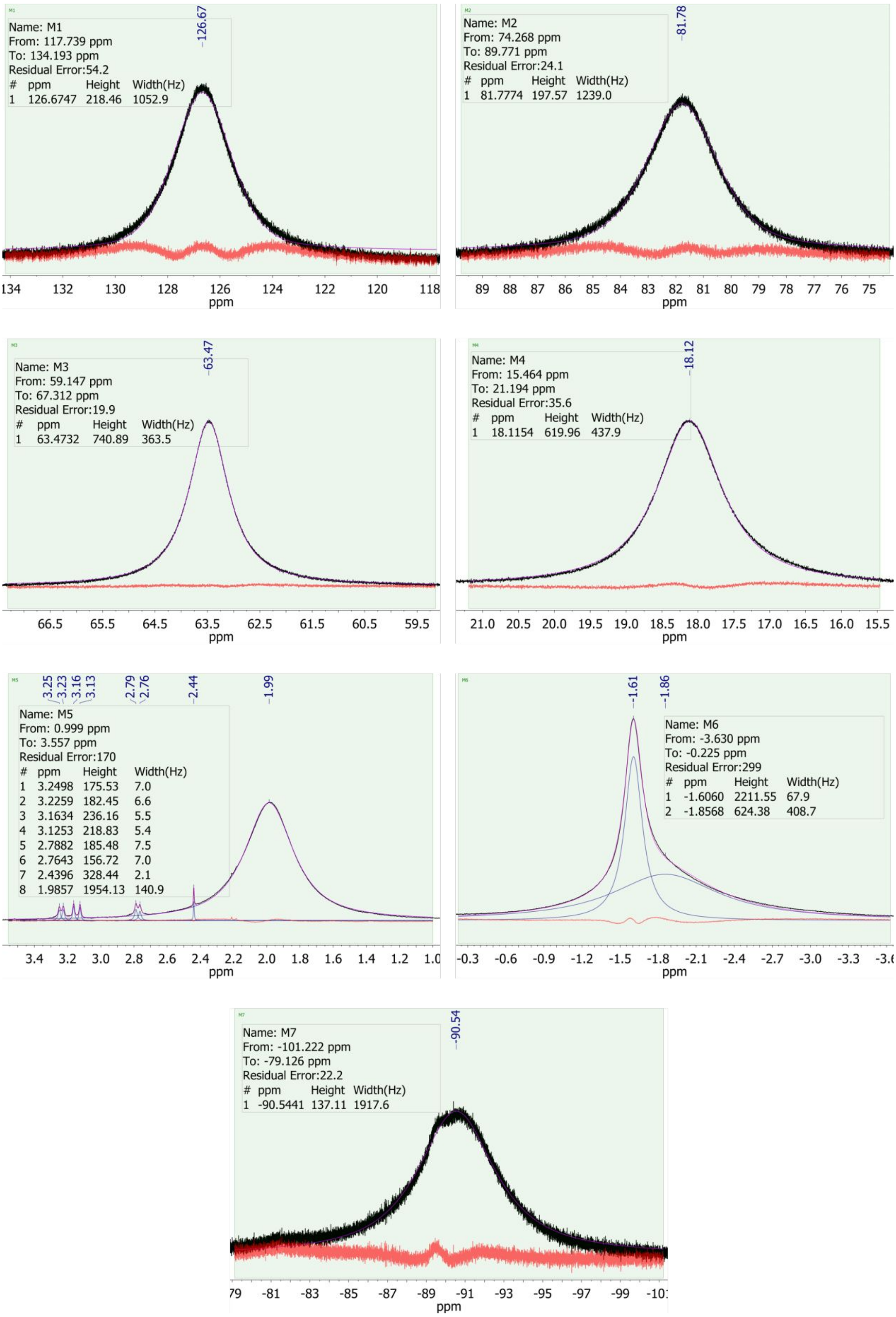

Figure S15. Fits of the ^{1}H NMR signals of Dy(PYTA)] to Lorentzian functions and linewidths. The diamagnetic region shows small signals due to a slight ligand excess (2.3-3.5 ppm) that have been deconvoluted.

Table S2: Crystal data and structure refinement for [Dy(CB-TE2PA)](PF_6)·2.5H_2O.

Empirical formula	$C_{52}H_{78}Dy_2F_{12}N_{12}O_{13}P_2$
Molecular weight MW	1694.20
Crystal system	Orthorhombic
Space group	Pbca /n
a/Å	13.941(2)
b/Å	21.573(4)
c/Å	41.361(7)
Volume (Å^3)	12439(3)
Z	8
ρ_{calc} (g/cm^3)	1.809
μ (mm^{-1})	2.543
θ range	1.89º-26.53º
R_{int}	0.036
Measured reflections	97462
Independent reflections / unique (I >2σ (I))	12839 / 10808
Goodness-of-fit on F^2	1.276
R_1	0.0660
wR_2 (all data)	0.1435
Larg. diff. peak-/ hole (eÅ^{-3})	1.47 and -1.49

Table S3: Cartesian coordinates (Å) of the [Dy(NO3PA)] complex optimized with DFT.

C	-3.15650900	-0.67624900	-1.14979800
C	-4.52701400	-0.58726500	-1.37463200
H	-4.97920600	-1.22073900	-2.12699600
C	-5.26598300	0.32336800	-0.62249700
H	-6.33745100	0.41289500	-0.76198400
C	-4.60971700	1.13232600	0.30460500
H	-5.15131600	1.86576600	0.89011100
C	-3.23192100	0.98640200	0.46468400
C	-2.39571800	1.87901000	1.34538800
H	-1.99858400	2.68752300	0.72304200
H	-3.00228700	2.33327900	2.13910000
C	0.99469300	3.06870200	-1.14862900
C	1.75395500	4.21415100	-1.36733500
H	1.43059900	4.92434900	-2.11753800
C	2.91028600	4.39901700	-0.61244000
H	3.52122000	5.28456700	-0.74671600
C	3.28368800	3.42275300	0.31046600
H	4.18847800	3.52429900	0.89794700
C	2.47134300	2.29955200	0.46488900

C	2.83141600	1.12751400	1.34120100
H	3.33149300	0.38157500	0.71472700
H	3.53231300	1.42609700	2.13112600
C	2.16310200	-2.38160400	-1.16260200
C	2.78531500	-3.60649100	-1.38418200
H	3.55953500	-3.67531400	-2.13767000
C	2.37840400	-4.70388900	-0.62847500
H	2.84684800	-5.67207400	-0.76518400
C	1.34722000	-4.54819400	0.29727000
H	0.99045600	-5.38641900	0.88393000
C	0.77179700	-3.28740500	0.45485400
C	-0.42426000	-3.02060200	1.33218300
H	-1.31908200	-3.08102000	0.70445500
H	-0.51398400	-3.77837600	2.12094800
C	-0.28068000	2.08889400	2.49944800
H	-0.71807700	2.59852300	3.37011500
H	-0.07261800	2.84619500	1.74341700
C	1.01453600	1.40899800	2.93855500
H	1.73518300	2.18719500	3.21003800
H	0.84459300	0.82906800	3.84710900
C	1.96191900	-0.81237500	2.49457900
H	2.62397800	-0.69220600	3.36443600
H	2.51074700	-1.37020900	1.73562100
C	0.72476100	-1.59239500	2.93370800
H	1.03592600	-2.60688600	3.20312100
H	0.30832800	-1.15617100	3.84314400
C	-1.67383700	-1.30348500	2.49142300
H	-1.89870300	-1.94064700	3.35900500
H	-2.43108600	-1.49889200	1.73195200
C	-1.73435600	0.15627200	2.93571800
H	-2.76940600	0.39085200	3.20405500
H	-1.14979500	0.29722800	3.84638000
N	-2.53547800	0.07480600	-0.22780900
N	1.33429600	2.15227200	-0.22924600
N	1.20301600	-2.22525800	-0.23903700
N	-1.24541800	1.12909800	1.90410400
N	1.61136200	0.50336000	1.90351100
N	-0.35821700	-1.65268100	1.89843800
Dy	-0.00353100	0.00167000	-0.14975900
C	2.46428400	-1.13845200	-1.97807100
O	3.32491900	-1.18940800	-2.86278500
O	1.74858200	-0.12006500	-1.65877700
C	-2.23994000	-1.57855500	-1.95600600
O	-2.72418600	-2.28716300	-2.84542100
O	-1.00236000	-1.49829100	-1.62296800
C	-0.24095400	2.72614700	-1.96047400
O	-0.78104900	1.60621300	-1.63935100
O	-0.61677500	3.50512400	-2.84274100

Table S4: Cartesian coordinates (Å) of the [Dy(PYTA)]$^{-}$ complex optimized with DFT.

Dy	-0.00000200	-0.00000800	-0.00000200
C	3.28016100	-1.13773100	-0.19751000
C	4.67193900	-1.17340900	-0.22497200
H	5.18463300	-2.10877400	-0.41052800

C	5.37828200	0.00001600	0.00005300
H	6.46145200	0.00002000	0.00005200
C	4.67193000	1.17343300	0.22507800
H	5.18461500	2.10880300	0.41063300
C	3.28015100	1.13774300	0.19761600
C	2.48704400	2.41149800	0.31914800
H	3.06859100	3.15765600	0.87715400
H	2.34010000	2.79684400	-0.69288200
C	0.31968300	3.41392500	0.68581600
H	0.91330900	4.32870400	0.81464500
H	-0.45390600	3.42632300	1.45229700
C	-0.31965500	3.41430800	-0.68378600
H	-0.91325300	4.32917200	-0.81213300
H	0.45395200	3.42710700	-1.45024800
C	-2.48702000	2.41168700	-0.31762500
H	-3.06855500	3.15821000	-0.87515200
H	-2.34003300	2.79637100	0.69464700
C	-3.28014100	1.13786700	-0.19688700
C	-4.67192000	1.17357200	-0.22432000
H	-5.18460800	2.10905800	-0.40927900
C	-5.37826800	0.00001000	-0.00003800
H	-6.46143800	0.00001300	-0.00003100
C	-4.67192400	-1.17355800	0.22423500
H	-5.18461700	-2.10904100	0.40920100
C	-3.28014700	-1.13786200	0.19678600
C	-2.48702700	-2.41168300	0.31751700
H	-3.06858100	-3.15822500	0.87499900
H	-2.33999400	-2.79633400	-0.69476100
C	-0.31967700	-3.41431200	0.68376900
H	-0.91328300	-4.32917500	0.81208800
H	0.45389600	-3.42711300	1.45026400
C	0.31972000	-3.41392800	-0.68580500
H	0.91335500	-4.32870500	-0.81460600
H	-0.45383600	-3.42633200	-1.45232000
C	2.48706000	-2.41148900	-0.31904300
H	3.06863100	-3.15766200	-0.87700500
H	2.34007000	-2.79680700	0.69299100
C	1.22258200	1.96632800	2.36082000
H	1.58107900	2.84607500	2.90962700
H	1.89955100	1.13138900	2.54944100
C	-0.17331000	1.56555300	2.85838800
C	-1.22264700	1.96770900	-2.35960800
H	-1.58124300	2.84775200	-2.90787300
H	-1.89956200	1.13282400	-2.54866600
C	0.17325100	1.56731200	-2.85746100
C	-1.22273400	-1.96771800	2.35955600
H	-1.58135900	-2.84775900	2.90780500
H	-1.89964800	-1.13282600	2.54859100
C	0.17315500	-1.56735200	2.85745900
C	1.22268800	-1.96633700	-2.36077800
H	1.58118600	-2.84609600	-2.90956500
H	1.89968500	-1.13141700	-2.54937700
C	-0.17316600	-1.56551600	-2.85841500
N	2.60036100	0.00000400	0.00005300
N	1.15973900	2.21506600	0.91170000

N	-1.15973100	2.21559200	-0.91033600
N	-2.60035000	0.00000100	-0.00005400
N	-1.15976200	-2.21559600	0.91028700
N	1.15978200	-2.21506700	-0.91165800
O	-0.93363800	1.02669500	1.98206300
O	0.93345300	1.02760900	-1.98154800
O	0.93338700	-1.02761900	1.98158900
O	-0.93355300	-1.02672000	-1.98210200
O	0.46984500	-1.79030400	4.04227200
O	-0.46987300	1.78734100	4.04345400
O	0.47004000	1.79038300	-4.04222800
O	-0.46970700	-1.78737700	-4.04347300

Table S5: Cartesian coordinates (Å) of the $[Dy(CB\text{-}TE2PA)]^+$ complex optimized with DFT.

Dy	0.00073100	0.07900900	0.00465200
C	0.85993100	-1.99842100	-2.30956600
C	2.06529400	-2.10637100	-1.40423200
C	3.13269000	-2.95616800	-1.64788800
H	3.11494900	-3.59872900	-2.51696100
C	4.20388700	-2.93977400	-0.75931500
H	5.06246700	-3.57872500	-0.92370700
C	4.16648800	-2.08993100	0.33958700
H	4.98584100	-2.05398200	1.04555600
C	3.04833400	-1.28021600	0.52709300
C	2.85789300	-0.41110000	1.73424200
H	3.82404000	-0.14251500	2.17437500
H	2.29982300	-0.99707200	2.46673700
C	2.89010100	1.78899900	0.68687800
H	3.94155600	1.49210200	0.72351300
H	2.83545800	2.75248700	1.19152200
C	2.49867900	1.93126800	-0.78235900
H	3.09149000	2.74521400	-1.21951900
H	2.74560700	1.02435700	-1.33449100
C	0.77430200	2.22729600	-2.44587200
H	1.08098500	1.26602500	-2.86394300
H	1.41619500	2.99849400	-2.88980600
C	-0.67345000	2.52394800	-2.84092600
H	-1.04801600	3.40621200	-2.31869900
H	-0.63908500	2.81882800	-3.89355200
C	-1.66693600	1.36673600	-2.79447700
H	-2.57972300	1.67622100	-3.31901900
H	-1.23820900	0.52656900	-3.34049300
C	-2.87788100	1.80711500	-0.68483500
H	-3.93116700	1.51667800	-0.72251700
H	-2.81761100	2.77176400	-1.18669100
C	-2.48635600	1.94302100	0.78494300
H	-3.07589100	2.75760300	1.22525600
H	-2.73689300	1.03461900	1.33318900
C	-0.75985800	2.22672100	2.44946200
H	-1.07289500	1.26663100	2.86577800
H	-1.39663600	3.00082100	2.89558900
C	0.68996000	2.51322200	2.84513100
H	0.65732700	2.80566800	3.89847900
H	1.07006300	3.39436100	2.32506800

C	1.67673400	1.35021600	2.79633800
H	2.59125600	1.65312300	3.32151800
H	1.24325600	0.51107800	3.34025500
C	-2.85751400	-0.38976000	-1.73772400
H	-2.30217200	-0.97702200	-2.47123500
H	-3.82204900	-0.11498600	-2.17777500
C	-3.05395600	-1.26097700	-0.53337800
C	-4.17914700	-2.06169700	-0.34897600
H	-4.99759900	-2.01596400	-1.05543300
C	-4.22492900	-2.91472100	0.74695500
H	-5.08911400	-3.54671900	0.90882900
C	-3.15448400	-2.94270500	1.63602200
H	-3.14258600	-3.58784500	2.50327800
C	-2.07965400	-2.10137100	1.39583000
C	-0.87559900	-2.00750700	2.30423100
C	0.68929800	3.48985700	-0.34802700
H	0.70372700	4.28339800	-1.10166200
H	1.46734500	3.74159200	0.36459700
C	-0.66817400	3.49353000	0.35405600
H	-0.67765100	4.28575200	1.10916700
H	-1.44481300	3.75126300	-0.35793900
N	2.03382600	-1.29115000	-0.34119500
N	2.05742100	0.81087400	1.45354400
N	1.04601000	2.19029300	-0.97996700
N	-2.05029500	0.82624300	-1.45321900
N	-1.03249000	2.19508000	0.98346600
N	-2.03953900	-1.28301300	0.33500900
O	0.01204600	-1.09792800	-1.94092600
O	0.75978500	-2.72682700	-3.29560000
O	-0.01912300	-1.11310900	1.94041000
O	-0.78345400	-2.74056000	3.28750200
